# Biallelic variants in Plexin B2 (*PLXNB2*) cause amelogenesis imperfecta, hearing loss and intellectual disability

**DOI:** 10.1136/jmg-2023-109728

**Published:** 2024-03-08

**Authors:** Claire E L Smith, Virginie Laugel-Haushalter, Ummey Hany, Sunayna Best, Rachel L Taylor, James A Poulter, Saskia B Wortmann, Rene G Feichtinger, Johannes A Mayr, Suhaila Al Bahlani, Georgios Nikolopoulos, Alice Rigby, Graeme C Black, Christopher M Watson, Sahar Mansour, Chris F Inglehearn, Alan J Mighell, Agnès Bloch-Zupan

**Affiliations:** 1 Institute of Medical Research, St James’s University Hospital, University of Leeds Faculty of Medicine and Health, Leeds, UK; 2 Institut de Génétique et de Biologie Moléculaire et Cellulaire (IGBMC), INSERM U1258, CNRS-UMR7104, Université de Strasbourg, Strasbourg, France; 3 Yorkshire Regional Genetics Service, Leeds Teaching Hospitals NHS Trust, Leeds, UK; 4 Manchester Centre for Genomic Medicine, St Mary's Hospital, Manchester University NHS Foundation Trust, Manchester, UK; 5 Division of Evolution and Genomic Sciences, Manchester Academic Health Science Centre, Faculty of Biology, Medicine and Health, School of Biological Sciences, University of Manchester, Manchester, UK; 6 EMQN CIC, Manchester, UK; 7 Department of Paediatrics, University Children's Hospital, Salzburger Landesklinken (SALK) and Paracelsus Medical University, Salzburg, Austria; 8 Amalia Children's Hospital, Radboudumc, Nijmegen, The Netherlands; 9 Dental & OMFS Clinic, Al Nahdha Hospital, Government of Oman Ministry of Health, Muscat, Oman; 10 Institute for Fundamental Biomedical Research, BSRC Alexander Fleming, Vari, Greece; 11 School of Dentistry, University of Leeds Faculty of Medicine and Health, Leeds, UK; 12 North East and Yorkshire Genomic Laboratory Hub, Central Lab, St James's University Hospital, Leeds Teaching Hospitals NHS Trust, Leeds, UK; 13 Lymphovascular Research Unit, Molecular and Clinical Sciences Research Institute, St George's Hospital, University of London, London, UK; 14 SW Thames Regional Centre for Genomics, St George's University Hospitals NHS Foundation Trust, London, UK; 15 Faculté de Chirurgie Dentaire, Université de Strasbourg, Strasbourg, France; 16 Centre de référence des maladies rares orales et dentaires O-Rares, Filière Santé Maladies rares TETE COU, European Reference Network CRANIO, Pôle de Médecine et Chirurgie Bucco-dentaires, Hôpital Civil, Hôpitaux Universitaires de Strasbourg (HUS), Strasbourg, France

**Keywords:** Dentistry, Dentistry, Genetic Research, Genetics, Genetics, Medical

## Abstract

**Background:**

Plexins are large transmembrane receptors for the semaphorin family of signalling proteins. Semaphorin-plexin signalling controls cellular interactions that are critical during development as well as in adult life stages. Nine plexin genes have been identified in humans, but despite the apparent importance of plexins in development, only biallelic *PLXND1* and *PLXNA1* variants have so far been associated with Mendelian genetic disease.

**Methods:**

Eight individuals from six families presented with a recessively inherited variable clinical condition, with core features of amelogenesis imperfecta (AI) and sensorineural hearing loss (SNHL), with variable intellectual disability. Probands were investigated by exome or genome sequencing. Common variants and those unlikely to affect function were excluded. Variants consistent with autosomal recessive inheritance were prioritised. Variant segregation analysis was performed by Sanger sequencing. RNA expression analysis was conducted in C57Bl6 mice.

**Results:**

Rare biallelic pathogenic variants in plexin B2 (*PLXNB2*), a large transmembrane semaphorin receptor protein, were found to segregate with disease in all six families. The variants identified include missense, nonsense, splicing changes and a multiexon deletion. *Plxnb2* expression was detected in differentiating ameloblasts.

**Conclusion:**

We identify rare biallelic pathogenic variants in *PLXNB2* as a cause of a new autosomal recessive, phenotypically diverse syndrome with AI and SNHL as core features. Intellectual disability, ocular disease, ear developmental abnormalities and lymphoedema were also present in multiple cases. The variable syndromic human phenotype overlaps with that seen in *Plxnb2* knockout mice, and, together with the rarity of human *PLXNB2* variants, may explain why pathogenic variants in *PLXNB2* have not been reported previously.

**WHAT IS ALREADY KNOWN ON THIS TOPIC**

- Plexins are large transmembrane proteins that act as receptors for the semaphorin family of signalling proteins. Semaphorin-plexin signalling controls cellular interactions that are critical during development as well as in adult life stages. Nine plexin genes have been identified in humans, but despite the apparent importance of plexins in development, only biallelic *PLXND1* and *PLXNA1* variants have so far been associated with Mendelian genetic disease.

**WHAT THIS STUDY ADDS**

- We identify rare biallelic pathogenic variants in *PLXNB2* as a cause of a new autosomal recessive, phenotypically diverse syndrome with amelogenesis imperfecta and sensorineural hearing loss as core features. Intellectual disability, ocular disease, ear developmental abnormalities and lymphoedema were also present in multiple cases. The variable syndromic human phenotype overlaps with that seen in *Plxnb2* knockout mice, and, together with the rarity of human *PLXNB2* variants, may explain why pathogenic variants in *PLXNB2* have not been reported previously.

**HOW THIS STUDY MIGHT AFFECT RESEARCH, PRACTICE OR POLICY**

- Individuals presenting with amelogenesis imperfecta and sensorineural hearing loss should be screened for mutations in *PLXNB2* and tested for other features of the syndrome. *PLXNB2* should be added to amelogenesis imperfecta, deafness and intellectual disability gene panels to improve mutation detection rates. Affected families should receive appropriate genetic counselling.

## Introduction

Development is a cascade of highly dynamic, time-critical, complex processes involving many signalling molecules and receptors that act to regulate cell proliferation, migration, adhesion and differentiation. This results in the formation of complex tissues, which further organise to effect organogenesis. Plexins are large transmembrane proteins that act as receptors for the semaphorin family of signalling proteins. Semaphorin-plexin signalling controls cellular interactions that are critical during development as well as in adult life stages (reviewed by Perälä *et al*).^1^ Semaphorin signalling modulates changes to both actin and microtubule organisation and therefore to the overall cytoskeleton, cell morphology, cell adhesion and cell motility (reviewed in Alto and Terman^2^).

The plexin gene family was originally identified in humans, and members were grouped according to the domain structure of the encoded proteins.^3 4^ Nine genes have been identified in both humans and mice, with class A plexins consisting of four genes (A1–A4), class B of three (B1–B3), and class C (C1) and class D (D1) of one each.^1 4^ Plexin family members share a common structure, with extracellular and intracellular portions. The extracellular portions contain a sema domain that binds with semaphorin ligands to activate signalling, as well as two or three PSI (plexin, semaphorin and integrin) domains and two or three glycine-proline rich IPT (immunoglobulin, plexin and transcription factor) domains. The intracellular portions are highly conserved^5^ and contain two R-Ras GAP motifs and one set of Plexin Rho-GTPase Association Motifs.

Class B plexins have an additional intracellular C-terminal PSD95, DLG1 and ZO1 (PDZ) interaction domain^6^ and an extracellular cleavage site for proprotein convertases.^1^ Plexin B2 (PLXNB2) participates in axonal guidance and cell migration.^7^ It is expressed widely but it also demonstrates a specific temporospatial pattern of expression throughout development, and its expression is distinct from that of other plexins, suggesting non-redundancy.^8^ RNA transcripts are detectable in mice from early fetal stages to adulthood within the brain.^9^ In situ hybridisation in E14 mouse embryos revealed high expression within several regions of the central nervous system, including many regions of the brain and retina.^8^ Expression was also high in the developing tooth bud, oral epithelium and in cartilage, with lower expression also detected in the cochlea, lung, kidney, epidermis and intestine.^8^ The exact role of PLXNB2 in tooth development is currently unknown, but semaphorins and plexin B1 have been found to be important for innervation of tooth buds^10^ and for dental stem cell migration ex vivo.^11^

*Plxnb2* knockout mice (*Plxnb2* ^-/-^) vary in phenotype, depending on their genetic background. *Plxnb2* ^-/-^ mice produced on inbred backgrounds did not survive gestation.^12 13^ The majority developed exencephaly, reflecting the importance of PLXNB2 activity for neural tube closure and potentially also its influence on the actin cytoskeleton. Other defects noted included abnormal development of the dentate gyrus, defects in cerebellar foliation and lamination, retarded development of the olfactory bulb and impaired neuronal proliferation. In contrast, when the same pathogenic variant was introduced into outbred CD1 mice, neural tube closure defects were less common, and after four generations, around 30% of *Plxnb2* ^-/-^ mice were viable and fertile. Despite the knockout mice having no obvious behavioural or motor defects, their cerebella were smaller and major brain foliation defects were still present.^12^ Heterozygous *Plxnb2* ^+/-^ mice had no apparent abnormalities.

Human *PLXNB2* variants (MIM*604293) and aberrant PLXNB2 expression have been associated with lung cancer,^14 15^ acute myeloid leukaemia,^16^ amyotrophic lateral sclerosis,^17^ glioblastoma,^18^ autism spectrum disorders with regression,^19^ psoriasis^20^ and first trimester euploid miscarriage.^21^ In contrast, despite the apparent importance of plexins in development, only biallelic *PLXND1* (MIM*620282) and *PLXNA1* (MIM*601055) variants have so far been associated with Mendelian genetic disease in humans. *PLXND1* variants cause multiple types of congenital heart defects (MIM#620294).^22^ *PLXNA1* variants cause Dworschak-Punetha neurodevelopmental syndrome which includes speech regression, autistic features and hyperactivity, variable sensorineural hearing loss (SNHL), and ocular, brain, facial and skin abnormalities (MIM#619955).^23^ The same authors also suggested that pathogenic variants in other plexins may be embryonic lethal or may cause a range of phenotypes that have not yet been recognised as part of one syndrome.^23^

Here we describe six families with probands carrying rare biallelic *PLXNB2* variants. Affected individuals manifest a complex, variable syndromic phenotype, the core features of which appear to be SNHL and amelogenesis imperfecta (AI), with intellectual disability also present in most cases.

## Materials and methods

### Patients

Affected individuals and family members were recruited in accordance with the principles outlined by the Declaration of Helsinki, with local ethical approval. Clinical evaluation captured disease features as part of routine patient care. Genomic DNA was obtained from venous blood samples using a salt-based extraction protocol, or from saliva using Oragene DNA Sample Collection Kits (DNA Genotek, Ottawa, Ontario, Canada), as detailed in the manufacturer’s instructions.

### Sequencing and analysis

Individuals were recruited and genomic DNA was subjected to SNP genotyping, exome or genome sequencing at different institutions. Sequencing and analysis methods for each family can be found in the online supplemental materials and methods. In summary, variants identified in next generation short-read sequencing data were filtered to exclude all changes other than missense, frameshift or stop variants, exonic insertion/deletions or variants located at splice consensus sites (up to 8 bp within introns or 3 bp within exons away from splice junctions). Synonymous variants outside of the splice region were discarded. Variants in the Genome Aggregation Database (gnomAD) (v2.2.1)^24^ were excluded if present at a global minor allele frequency of 1% or higher. Variants were also filtered based on the mode of inheritance. In families known to be consanguineous, homozygous variants were prioritised. Population-specific high-frequency variants and platform artefacts were excluded by removing variants also present in exomes of individuals of the same ethnicity without dental disease that had been sequenced using the same reagents and platform. Splicing prediction analysis was carried out using NetGene2 (v2.4.2)^25^ and Splice AI.^26^ CADD v1.6,^27^ REVEL,^28^ Polyphen-2 (HumVar model)^29^ and SIFT^30^ were used to assess each variant’s potential to be disease causing. CNVs were identified using ExomeDepth (v1.0.0).^31^ Variants were confirmed and segregation analysis was performed for all available family members by Sanger sequencing. Primer sequences used are shown in online supplemental table 1. Genomic coordinates are based on the GRCh37 human reference genome, the reference gene sequence used for *PLXNB2* is MANE Select transcript NM_012401.4 (ENST00000359337.9) and protein variant nomenclature for PLXNB2 is based on RefSeq protein NP_036533.2 (ENSP00000352288.4). The corresponding references used for *CRYBB3* are MANE Select transcript NM_004076.5 (ENST00000215855.7) and for CRYBB3 RefSeq protein NP_004067.1 (ENSP00000215855.2). All variants identified as part of this study were uploaded to ClinVar: SCV002822954–SCV002822961. In silico modelling of the effect of the variants on the PLXNB2 protein tertiary structure was completed using I-TASSER-MTD^32^ using the default parameters. The protein structures were visualised with UCSF Chimera.^33^

10.1136/jmg-2023-109728.supp1 Supplementary data

### Mouse tissue preparation

All animals were maintained in accordance with the French Ministry of Agriculture guidelines for the use of laboratory animals under study (SC67-218-37-IGBMC and APAFIS 3957-2016020516359388v1) and in accordance with the National Institutes of Health guidelines provided in the Guide for the Care and Use of Laboratory Animals. All methods and experimental procedures were reviewed and approved by an institutional safety committee.

Mouse embryos/fetuses were collected at E14.5, E16.5, E19.5 or on the day of birth and analysed as detailed in the online supplemental materials and methods.

## Results

**Figure 1:**
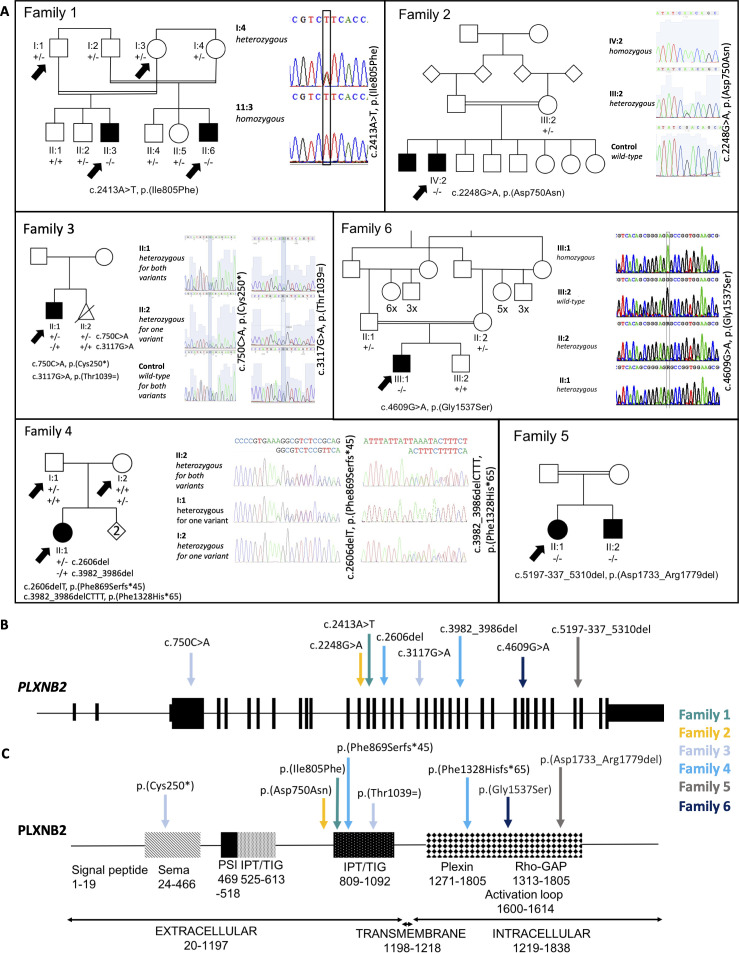
Pedigrees, Sanger sequencing and schematic diagram of the PLXNB2 protein. (A) DNA was available for all labelled individuals on each pedigree. Arrows indicate the individuals whose DNA was exome or genome sequenced. Affected status is as reported by the families for individuals for which DNA was not available. Sanger sequencing traces showing the segregation of each variant with disease in each family, except for Family 5, for which this is shown in online supplemental figure 9. The schematic diagram shows (B) the *PLXNB2* transcript (ENST00000359337.9, NM_012401.4; 6409 bp) and (C) the PLXNB2 protein (ENSP00000352288.4, NP_036533.2; 1838 amino acids), with the positions marked for the pathogenic variants identified in this study. IPT, immunoglobulin, plexin and transcription factor; PSI, plexin, semaphorin and integrin; TIG, transcription factor immunoglobin.

Initially, we recruited two consanguineous families, one Turkish (Family 1) and one Omani (Family 2) (figure 1). In Family 1, two male double first cousins have bilateral SNHL, intellectual disability, AI and severe myopia (online supplemental figure 1). In addition to the shared phenotype, one (II:3) also has bilateral cataracts and the other (II:6) has unilateral renal agenesis and pyloric stenosis. Analysis of GeneChip Human Mapping 250K *Nsp* SNP data from affected individuals II:3 and II:6 showed two homozygous regions encompassing chr7:34,080,354-41,760,000 and chr22:49,714,781-51,1756,26. Exome sequencing of II:3 and II:6 revealed a shared homozygous variant in *PLXNB2* within the region on chromosome 22, c.2413A>T, p.(Ile805Phe) (table 1) which segregates with disease in the family (online supplemental figure 2). It affects the extracellular portion of the protein, specifically the first cell surface receptor IPT domain, and is predicted to be damaging by all pathogenicity prediction software tested (online supplemental table 2). The residue affected is highly conserved (online supplemental figure 3) and the variant is absent from gnomAD. Individual II:3 was also found to carry variant c.388G>A, p.(Glu130Lys) in crystallin beta 3 (*CRYBB3*; MIM*123630) (online supplemental figure 4), which is likely to explain the bilateral cataracts observed in him (MIM#609741).^34^ Variants that passed population and pathogenicity prediction filters but were not investigated further are detailed in online supplemental table 3 for each family.

**Table 1:** The variants in *PLXNB2* identified in six families and the disease features observed

Patient (sex/age range)	Zygosity and variants (NM_012401.4, NP_036533.2)	Phenotype				
		Auditory	Dental	Developmental/neurological	Vision	Other
Family 1 II:3 (M/12–18)	Homozygous c.2413A>T: p.(Ile805Phe); c.2413A>T: p.(Ile805Phe)	SNHL	AI, conical permanent incisors	Global developmental delay, moderate intellectual disability	Myopia, horizontal nystagmus (congenital cataract due to *CRYBB3* variant)	Ear lobe skin blind-ended tracts
Family 1 II:6 (M/19–21)	Homozygous c.2413A>T: p.(Ile805Phe); c.2413A>T: p.(Ile805Phe)	SNHL; labyrinthine malformation	AI, conical permanent incisors	Global developmental delay, epilepsy, moderate intellectual disability	Severe myopia with scattered papillae, horizontal nystagmus, macular atrophy	Ear lobe skin blind-ended tracts, unilateral renal agenesis, pyloric stenosis, asthma, recurrent bronchitis, intrauterine growth retardation, finger pads, watch glass toenails, overweight
Family 2 IV:2 (M/12–18)	Homozygous c.2248G>A: p.(Asp750Asn); c.2248G>A: p.(Asp750Asn)	SNHL	AI	Intellectual disability	No obvious abnormality, not examined	
Family 3 II:1 (M/6–11)	Compound heterozygous c.750C>A: p.(Cys250*); c.3117G>A: p.(Thr1039=)	SNHL (mild) 500–4000 Hz	AI with hypoplasia	Normal	Developmental macular abnormality with pale fundus, attenuated blood vessels, high myopia, nystagmus, microcornea	Ear lobe skin blind-ended tracts, cleft palate, hypertelorism, keratopathy
Family 4 II:1 (F/19–21)	Compound heterozygous c.2606del: p.(Phe869Serfs*45); c.3982_3986del: p.(Phe1328His*65)	SNHL	AI; missing upper permanent lateral incisors	Normal	No obvious abnormality, not examined	Ear lobe skin blind-ended tracts, bilateral primary lower limb lymphoedema (onset aged 3), nevus, cellulitis
Family 5 II:1 (F/50–59)	Homozygous c.5197-337_5310del: p.(Asp1733_Arg1779del); c.5197-337_5310del: p.(Asp1733_Arg1779del)	SNHL	AI	Mild/moderate intellectual disability	No obvious abnormality, not examined	Bilateral primary lower limb lymphoedema
Family 5 II:2 (M/50–59)	Homozygous c.5197-337_5310del: p.(Asp1733_Met1770del); c.5197-337_5310del: p.(Asp1733_Met1770del)	SNHL	AI	Intellectual disability	No obvious abnormality, not examined	Unilateral lymphoedema of one foot
Family 6 III:1 (M/2–5)	Homozygous c.4609G>A: p.(Gly1537Ser); c.4609G>A: p.(Gly1537Ser)	Could not be assessed; no current indication of hearing loss	Clinical tooth failure (cause unclear); could not be assessed further	Profound intellectual disability, non-verbal, autistic features, hyperactive behaviour	Strabismus, no other obvious abnormality, could not be assessed	Mild generalised muscular hypotonia

Splicing prediction tools suggest that exon 35 is entirely skipped leading to p.(Asp1733_Arg1779del) instead of p.(Asp1733_Met1770del) as would be predicted from the proportion of the gene deleted. Age ranges are shown for individuals to maintain anonymity. Age of onset was from birth unless otherwise stated. Variants are based on genome build GRCh37 and *PLXNB2* transcript ENST00000359337.9, NM_012401.4 and PLXNB2 protein ENSP00000352288.4, NP_036533.2.

AI, amelogenesis imperfecta; SNHL, sensorineural hearing loss.

In Family 2, two Omani brothers born of a first cousin union were found to have bilateral SNHL, intellectual disability and AI (table 1, figure 1 and online supplemental figure 5). Exome sequencing of one affected brother revealed a homozygous missense variant in *PLXNB2*, c.2248G>A, p.(Asp750Asn). This replaces a charged residue with an uncharged one, affecting a highly conserved residue in the extracellular portion of the protein (online supplemental figure 3). SIFT predicts the variant to be deleterious and it was not identified in gnomAD (online supplemental table 2).

**Figure 2:**
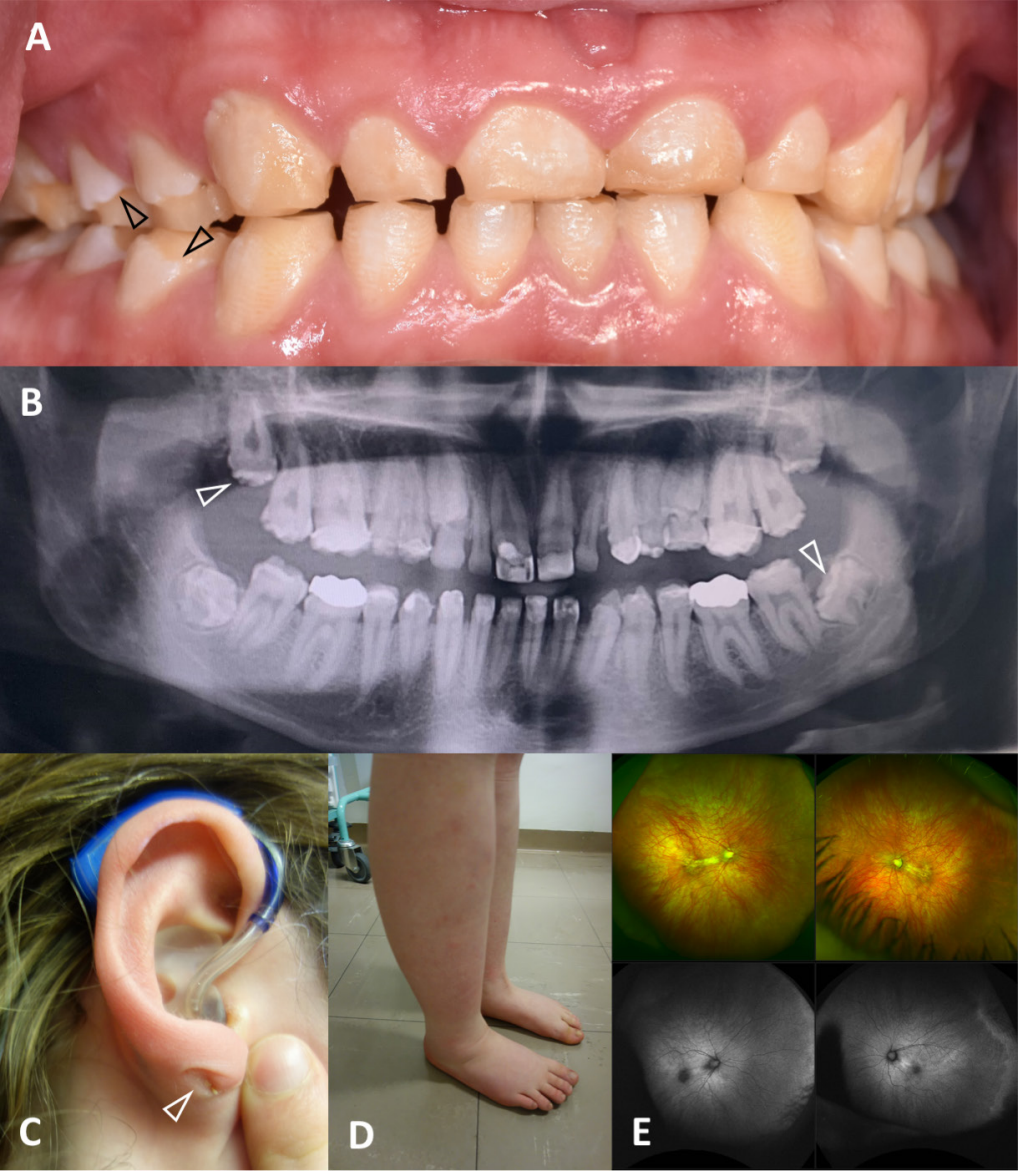
Clinical images that are illustrative of the shared and variable clinical features for affected individuals. (A) The anterior clinical photograph showing primary teeth, the posterior teeth have changes consistent with loss of enamel (arrowheads) due to fracturing, whereas the enamel of the anterior teeth is developmentally thin and optically abnormal (Family 3 II:1). (B) Panoramic radiograph of the adult permanent dentition illustrates that the enamel is more radiodense than the supporting dentine, but with a variably reduced enamel volume and irregular occlusal cusp morphology (arrowhead) consistent with amelogenesis imperfecta (AI) characterised by enamel that is hypomineralised with variable hypoplasia (Family 4 II:2). (C) Sensorineural hearing loss (SNHL) was typical, with provision of hearing aids in childhood (Family 4 II:2). Blind-end skin tracts involving the skin of the ears or adjacent tissues (arrowhead) were observed in at least 3/6 families. (D) Lower limb lymphoedema was observed in two families (Families 4 and 5, with Family 4 illustrated). (E) Fundus autofluorescence images (Family 3 II:1) illustrate a developmental macular abnormality with pale fundus and attenuated blood vessels. The individual also has high myopia, nystagmus and microcornea.

Family 3 was identified independently via the UK Inherited Retinal Dystrophy Consortium. They are a white British family with an affected male child born with facial clefting, who also presented with nystagmus shortly after birth. In middle childhood, he was diagnosed with retinal dystrophy, high myopia, microcorneas and mild keratopathy. He also has mild bilateral SNHL for sounds ranging from 500 to 4000 Hz, and AI (table 1, figures 1 and 2 and online supplemental figure 6). Exome sequencing revealed biallelic compound heterozygous variants in *PLXNB2*, one a nonsense variant, c.750C>A, p.(Cys250*), and one synonymous variant altering the final base of the splice donor site of exon 19, predicted to affect splicing, c.3117G>A, p.(Thr1039=). Analysis with splice prediction tools Splice AI^26^ and NetGene2^25^ predicted loss of the donor site, suggesting that some of intron 19 may be retained in the mature transcript (online supplemental table 4, online supplemental figure 7). Neither variant was identified in gnomAD (online supplemental table 2). A second fetus, who presented with clefting, was electively aborted. Genotyping showed that the fetus was heterozygous for the nonsense *PLXNB2* variant only.

Next, we searched the UK 100,000 Genomes dataset^35^ for patients with biallelic variants in *PLXNB2* and a similar phenotype. We identified one affected white British female (Family 4) with SNHL, AI, lower limb lymphoedema and cellulitis (table 1, figures 1 and 2, online supplemental figure 8). She carries biallelic compound heterozygous frameshift variants c.2606delT, p.(Phe869Serfs*45) and c.3982_3986delCTTT, p.(Phe1328Hisfs*65) in *PLXNB2* (online supplemental figure 9), both predicted to produce transcripts that are subject to nonsense mediated decay.^36^ Variant c.2606delT, p.(Phe869Serfs*45) has previously been identified in gnomAD as a heterozygous variant in one individual, suggesting an allele frequency of 4.024×10^–6^. Variant c.3982_3986delCTTT, p.(Phe1328Hisfs*65) was not present in gnomAD.

With increased understanding of the clinical presentation associated with biallelic *PLXNB2* variants, we identified Family 5 through further collaboration. Two affected siblings of Pakistani origin, born of a consanguineous union (figure 1), presented with deafness, AI, intellectual disability and lower limb lymphoedema (figure 2, table 1, online supplemental figures 10 and 11). Exome sequencing of II:1 and subsequent ExomeDepth analysis revealed a homozygous deletion spanning exons 34 and 35 (Reads ratio 0.0291, Bayes factor 18.3; online supplemental table 2). The breakpoints predicted in the exome sequence (online supplemental figure 12), chr22:50,715,085 and chr22:50,715,672, were confirmed by PCR, which also confirmed the deletion was present in II:2. This deletion is in-frame and is predicted to delete at least 38 amino acids (aa) (p.(Asp1733_Met1770del)) from the 1838aa protein, including all of exon 34 and part of exon 35. Splice prediction tool NetGene2 (v2.4.2) predicts that exon 35 will be skipped entirely (online supplemental table 5), resulting in an in-frame deletion of 47aa, p.(Asp1733_Arg1779del), suggesting that an abnormal PLXNB2 protein may be produced. The deleted region is part of the Rho-GAP catalytic domain critical to the protein’s function, but the deletion would leave the catalytic arginine residues at 1395, 1396 and 1691 intact.

Using GeneMatcher,^37^ we identified one further patient with biallelic *PLXNB2* variants and an overlapping disease phenotype. Family 6 is of Iraqi origin. One affected male was born of first cousin consanguineous parents (figure 1). In early childhood, he has severe developmental delay and autistic features, and his tooth enamel shows evidence of severe damage from a limited visual inspection (table 1, online supplemental figure 13). However, it was not possible to carry out a detailed dental examination or to obtain dental radiographs. The presence of AI, as opposed to severe caries, could therefore not be confirmed, and testing for SNHL and eye disease was not possible. On exome sequencing, the affected individual was found to carry a homozygous *PLXNB2* variant c.4609G>A, p.(Gly1537Ser), which affects the highly conserved Rho-GAP domain that lies within the intracellular portion of PLXNB2 and changes the residue from a non-polar to a polar residue (table 1, online supplemental table 2, online supplemental figure 3). The residue is conserved in all species examined and this variant was not present in gnomAD.

We next used I-TASSER-MTD to try to assess the effect of each of the variants on the overall predicted structure of PLXNB2 (online supplemental figure 14). In silico predictions of WT PLXNB2 structure (panel A) and of these variants are likely to be of limited use due to the small percentage of PLXNB2 covered by known crystal structures and the lack of appropriate homologous protein structures on which to base the WT structure. The local structural changes for the missense variants (Asp750Asn, Ile805Phe and Gly1537Ser) are shown in panels B, C and D. There were minor changes in the solvent accessibility for Asp750Asn and Ile805Phe. For Ile805Phe, the mutant structure is characterised as undefined, in comparison to the WT strand structure. The model for the deletion Asp1733_Arg1779del shows extensive structural differences to the WT protein.

**Figure 3:**
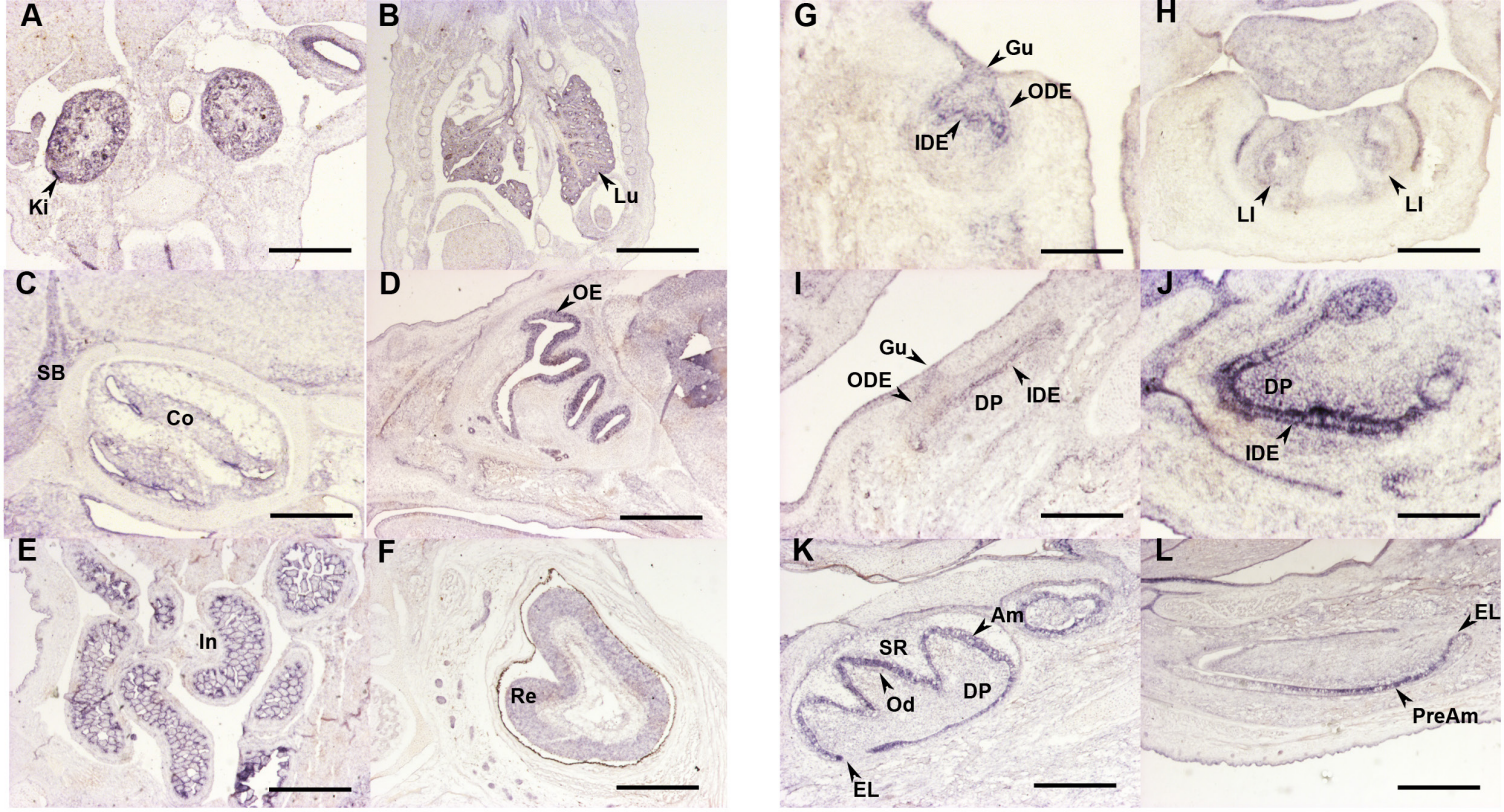
Analysis of mouse *Plxnb2* transcript distribution by in situ hybridisation. Selected sections illustrating *Plxnb2* expression features in the developing skull bone, sensorial organs and viscera are shown in the left side panels (A–F), whereas right side panels focus on incisor (G, I and K) and molar (H, J and L) tooth development. Developmental stages and section planes are: E14.5 frontal (A, B, G and H), E16.5 sagittal (C, D, E, I and J); E19.5 sagittal (F, K and L) sections. Scale bars: 25 µm (J); 40 µm (H and I); 50 µm (C and K); 60 µm (F and H), 80 µm (A, D, E and L); 150 µm (B and G). Am, ameloblasts; Co, cochlea; DP, dental papilla; EL, epithelial loop; Gu, gubernaculum; IDE, inner dental epithelium; In, intestine; Ki, kidney; LI, lower incisor; Lu, lung; Od, odontoblasts; ODE, outer dental epithelium; OE, olfactory epithelium; Re, retina; SB, skull bone; SR, stellate reticulum.

To gain insight into *Plxnb2* expression in mice, we performed in situ hybridisation using a digoxigenin-labelled antisense riboprobe generated from the same DNA template as previously used by the EURExpress consortium (www.eurexpress.org) (online supplemental figure 15). Mouse embryos were analysed at E14.5, E16.5 and E19.5. *Plxnb2* expression was detected in the kidney and lung (figure 3A,B). Discrete expression was observed in the developing inner ear (cochlea: figure 3C). Expression was also detected in olfactory epithelium and retina (figure 3D,F) and in the small and large intestines (figure 3E).

We also interrogated the GTEx portal^38^ (https://gtexportal.org/) to determine the expression of *PLXNB2* in human tissues. This showed that there was relatively high expression of *PLXNB2* in the cerebellum and cerebellar hemisphere compared with other regions of the brain. There was also high expression in the kidney, salivary gland, ovary, prostate, spleen and thyroid, among other tissues (online supplemental figure 16).

Since all individuals studied had AI or were suspected to have AI, we investigated the expression of *Plxnb2* in the mouse during various stages of tooth development (figure 3G,I,K lower molar and figure 3H,J,L lower incisor). *Plxnb2* transcripts were observed at E14.5 in the epithelial tissues of the developing teeth, at E16.5 in the epithelial and mesenchymal compartments of the incisor and in the epithelial part of the molar. At E19.5, labelling was observed in differentiating ameloblasts.

## Discussion

Here we describe biallelic pathogenic variants in *PLXNB2* in six families of diverse ethnicity with individuals affected by a variable syndromic phenotype. Four of these are consanguineous families with affected individuals homozygous for an extremely rare variant that has almost certainly been passed down both branches of the family, and one includes affected cousins, demonstrating significant cosegregation of the disease with biallelic *PLXNB2* variants. This syndrome has AI and SNHL as core symptoms, and intellectual disability, lower limb lymphoedema, ocular abnormalities and a variety of other conditions are also seen in some, but not all, cases. Given the varied and complex roles of PLXNB2 in development,^1^ it seems unsurprising that biallelic variants cause a syndromic disease phenotype. The variants all have pathogenicity scores indicating that they are predicted to be deleterious (online supplemental table 2). The case series we have accumulated, together with published evidence of an overlapping condition in *Plxnb2* knockout mice, provides compelling evidence for biallelic variants in *PLXNB2* as the cause of this recessively inherited condition.

All variants implicated are extremely rare, with all but one absent in gnomAD (v2.1.1), which contains approximately 124 000 individuals with good quality sequence across *PLXNB2*. The rarity of these variants, together with the constraint metrics available in gnomAD (o/e=0.19, pLI=0.99),^39^ suggests that *PLXNB2* loss-of-function variants are not well tolerated and affect viability. However, the identification of an individual who may entirely lack PLXNB2 (Family 4 II:1) seems to contradict this. This is consistent with observations in *Plxnb2* knockout mice, where homozygosity for the knockout allele was lethal on one genetic background but viable on another.^12 13^ The *PLXNB2* variants identified herein include missense, frameshift and splice variants, a premature termination codon and a deletion spanning two exons, which are all observed to be protein-damaging variants.

One possible interpretation of these findings is that all the observed human variants have the effect of being functional knockouts, and that, as observed in mouse models, the viability of such embryos is determined by the genetic background. Genetic background might also explain the highly variable phenotypes observed in different cases, with only AI and SNHL as consistent features. Pathogenic variants in *PLXNA1* cause an overlapping and similarly varying range of phenotypes to pathogenic variants in *PLXNB2*.^23^ The impact of genetic background on variation in disease phenotype, severity and survival has been noted for one *Plxnb2* ^-/-^ mouse model,^12^ which suggests that other cosegregating variants may affect disease range and severity. This may suggest that, in spite of the distinct patterns of plexin expression,^8^ other plexins can sometimes partially compensate for loss of PLXNB2 or PLXNA1 to allow developmental processes vital to life to proceed. However, to prove such an effect would require the study of a large cohort of cases, and as yet no specific variants have been implicated in phenotype variation in either of these syndromes. Given that this cohort consists of only eight individuals with six different biallelic genotypes, the power to detect any genotype-phenotype correlation in this study is very limited.

Alternatively, the *PLXNB2* variants and/or genotypes in the families described herein may each have unique effects on PLXNB2 function. Variants could cause partial loss of function through hypomorphic alleles, with expression of the normal transcript reduced but not abolished,^40^ or may act as ‘gain-of-function’ alleles, creating a protein with altered or enhanced function or inappropriate persistence within the cell. Consistent with this hypothesis, the affected cousins in Family 1 and affected siblings in Family 5, each having the same genotype, have remarkably similar phenotypes (table 1).

The missense variants identified in this study affect both the extracellular (p.(Ile805Phe) and p.(Asp750Asn)) and intracellular portions of the protein (p.(Gly1537Ser)), with no particular region or domain specifically affected by the variants identified in this study. In order to assess the effects of the variants on protein structure, we attempted to model the changes using I-TASSER-MTD.^32^ However, due to the lack of homologous structures available for the majority of the PLXNB2 protein, including the regions affected by the variants, the accuracy of the modelling is likely to be low. The variants identified herein could be altering the binding of PLXNB2 to semaphorins via the sema domain, the subsequent homodimerisation of PLXNB2 on binding semaphorin, catalytic activity via the GAP domain and interaction with GTPases, or the ability to interact with other proteins and to effect other types of downstream signalling.^41^ Further investigation will be required to better understand the effects of specific variants and the basis of variation on phenotype in the condition caused by *PLXNB2* variants.

Heterozygous carriers of the variants identified in these families appear in general to be unaffected by disease, although one fetus (Family 3 II:2) did have facial clefting and carried a heterozygous nonsense *PLXNB2* variant (c.750C>A, p.(Cys250*)). It is unknown whether this fetus would have developed other clinical features similar to their sibling. It is possible that another variant could be partially or entirely responsible for this particular phenotype rather than the *PLXNB2* variant. In individuals with *PLXNA1* pathogenic variants, disease has been observed with both biallelic and particular de novo heterozygous variants.^23^

The core phenotypes observed for the individuals carrying pathogenic *PLXNB2* variants reflect negative impacts on the development or function of the cochlea and ameloblasts. This led us to consider whether other plexins are expressed in the cochlea and inner enamel epithelium at a similar time to PLXNB2, or whether PLXNB2 is expressed alone in these tissues, excluding the possibility of partial compensation by a closely related protein. Our investigation of the expression of *Plxnb2* transcripts within the dental tissues of murine embryos gave similar results to the expression patterns previously detailed by Perälä *et al*.^8^ Their analysis of the expression of plexins in murine embryos using in situ hybridisation revealed that *Plxnb2* transcripts are present in the brain, retina, cochlea and tooth bud at E14.^8^ *Plxnb2* is the only plexin to be expressed at this timepoint in all four tissues, although the expression of *Plxnd1* was not examined in this study and the expression of other plexins, most notably *Plxnb1*, does overlap that of *Plxnb2* in many tissues. *Plxnb2*, *Plxna2* and *Plxna3* transcripts are all expressed in the cochlea, although the relative levels of expression of each were not determined. Similarly, *Plxnb1* and *Plxnb2* transcripts were detected at relatively high levels at E14 in the oral epithelium and tooth bud, but other plexins were also detected at lower levels of expression. *Plxnb2* was shown to be expressed at relatively high levels in the inner enamel epithelium at E15, with expression sustained until at least E16. These findings suggest that PLXNA2, PLXNA3 and PLXNB1 are coexpressed in affected tissues at relevant timepoints, but are unlikely to be able to fully compensate for the loss of PLXNB2.

The variable phenotype and extremely low population frequency of the variants reported in this study may be the reasons why pathogenic variants in *PLXNB2* have not previously been reported as causing syndromic disease in humans. We suggest that the tooth enamel phenotype, AI, is a consistent, but potentially easily missed feature that flags this genetic diagnosis. AI is a heterogeneous group of genetic conditions characterised by a deficit in enamel quantity and/or quality affecting all teeth of both dentitions.^42–44^ It can present as an isolated disease or can be part of more complex and diverse syndromes affecting other tissues and organs. Once formed and following tooth eruption, enamel has no capacity for cellular repair. Accordingly, AI provides a clear and persistent marker of abnormal development that is recognisable at an early age. However, due to the presence of neuronal deficits such as hearing loss and intellectual disability, AI might easily be overlooked or dismissed as dental caries due to suboptimal diet and/or poor dental hygiene. A differential diagnosis of AI as opposed to fluorosis or molar incisor hypomineralisation is also a possible confounding issue. Diagnosis of AI may therefore require a specialist paediatric dental professional. The disconnection between dental and general healthcare also presents barriers to diagnosis and has been flagged as problematic previously in the differential diagnosis of Usher and Heimler syndromes.^45^

The families presented have AI characterised by variable abnormalities of enamel volume (hypoplasia) and mineralisation (hypomineralised). This is consistent with the understanding of PLXNB2 function. Future laboratory investigation of enamel from affected individuals will give insight into the characteristics of the disruption to enamel rod morphology and mineralisation. It is unclear if the other dental morphological changes reported, including conical lateral incisors, missing lateral incisors, flattened occlusal surfaces and mild taurodontism, are consequent to PLXNB2 functional disruption or are coincidence in these families. As further families are described, the core features of the dental phenotype will become clearer and will be an important clinical indicator to consider *PLXNB2* further.

In conclusion, we identify biallelic pathogenic variants in *PLXNB2* as a cause of a new autosomal recessively inherited, phenotypically diverse syndrome including AI and SNHL as core symptoms, with intellectual disability, ocular disease, ear developmental abnormalities and lymphoedema also present in multiple cases.

## Data Availability

All data relevant to the study are included in the article or uploaded as supplementary information. Partial (anonymised) information can be provided on request.
