# Supplementary material for: Biallelic variants in Plexin B2 (PLXNB2) cause amelogenesis imperfecta, hearing loss and intellectual disability
Source: J Med Genet. 2024 Mar 8;61(7):689–98. doi: 10.1136/jmg-2023-109728 (PMC11228227; doi:10.1136/jmg-2023-109728)
Supplement: Supplementary data [file jmg-2023-109728supp001.pdf]

Biallelic variants in Plexin B2 (*PLXNB2*) cause amelogenesis imperfecta, hearing loss and intellectual disability.

Claire E L Smith[1*], Virginie Laugel-Haushalter[2*], Ummey Hany[1], Sunayna Best[1,3], Rachel L Taylor[4,5,6], James A. Poulter[1], The UK Inherited Retinal Disease Consortium, Genomics England Research Consortium, Saskia B Wortmann[7,8], René G Feichtinger[7], Johannes A Mayr[7], Suhaila Al Bahlani[9], Georgios Nikolopoulos[10], Alice Rigby[1,11], Graeme C Black[4,5], Christopher M. Watson[1,12], Sahar Mansour[13,14], Chris F Inglehearn[1], Alan J Mighell[11*] and Agnès Bloch-Zupan[2,15,16*]

[1]Leeds Institute of Medical Research, St James's University Hospital, University of Leeds, Beckett Street, Leeds. LS9 7TF. UK.
[2]Institut de Génétique et de Biologie Moléculaire et Cellulaire (IGBMC), INSERM U1258, CNRS-UMR7104, Université de Strasbourg, Illkirch, France.
[3]Yorkshire Regional Genetics Service, Leeds Teaching Hospitals NHS Trust, Leeds. LS7 4SA. UK.
[4]Division of Evolution and Genomic Sciences, Manchester Academic Health Science Centre, Faculty of Biology, Medicine and Health, School of Biological Sciences, University of Manchester, Manchester, M13 9NT. UK.
[5]Manchester Centre for Genomic Medicine, St. Mary's Hospital, Manchester University NHS Foundation Trust, Manchester. M13 9WL. UK.
[6]EMQN CIC, Pencroft Way, Manchester Science Park, Manchester. M15 6SE. UK.
[7]University Children's Hospital, Salzburger Landesklinken (SALK) and Paracelsus Medical University (PMU), Müllner Hauptstrasse 48, 5020 Salzburg, Austria.
[8]Amalia Children's Hospital, Radboud University Medical Center, Geert Grooteplein Zuid 10, 6525 GA Nijmegen, the Netherlands.
[9]Dental & O.M.F.S Clinic, Al Nahdha Hospital, Ministry of Health, Muscat. HGQJ+GV6. Oman.
[10]Institute for Fundamental Biomedical Research, BSRC Alexander Fleming, 16672 Vari, Attica, Greece.
[11]School of Dentistry, University of Leeds. LS2 9JT. UK.
[12]North East and Yorkshire Genomic Laboratory Hub, Central Lab, St. James's University Hospital, Leeds. LS9 7TF. UK.
[13]Lymphovascular Research Unit, Molecular and Clinical Sciences Research Institute, University of London St George's, London. SW17 0QT. UK.
[14]SW Thames Regional Genetics Service, St George's University Hospitals NHS Foundation Trust, London. SW17 0QT. UK.
[15]Faculté de Chirurgie Dentaire, Université de Strasbourg, 8 Rue Sainte Elisabeth, F-67000 Strasbourg, France.
[16]Centre de référence des maladies rares orales et dentaires O-Rares, Filière Santé Maladies rares TETE COU, European Reference Network CRANIO, Pôle de Médecine et Chirurgie Bucco-dentaires, Hôpital Civil, Hôpitaux Universitaires de Strasbourg (HUS), 1 place l'hôpital, 67000 Strasbourg, France.

* These authors contributed equally to the study.
Correspondence to:
Dr Claire E L Smith email C.E.L.Smith@leeds.ac.uk
Telephone (+44) 113 343 8445

Supplementary Materials and Methods.

Sequencing and analysis

Since individuals were recruited and sequenced by different institutions, the sequencing and filtering methods used vary and are detailed below.

Family 1

Regions of homozygosity were identified using the GeneChip® Human Mapping 250K *Nsp* assay (Affymetrix). Results were analysed using Affymetrix software. WES was performed by IntegraGen (Evry, France; https://integragen.com). WES and bioinformatic analysis followed the protocol previously described.[1] Briefly exons were captured using the SureSelect Human All Exon V5 + UTR 75 Mb reagent (Agilent Technologies, USA) and sequenced using an Illumina HiSeq 2000 sequencer (Illumina, USA). The Burrows-Wheeler Aligner (v7.12) was used to align reads to the Human genome reference sequence GRCh37. Variant calling was carried out using the HaplotypeCaller module of Genome Analysis Toolkit (GATK, v.3.4.36). Copy number variants were called using CANOES.[2] The variants were filtered to exclude variants with an allele frequency of more than 1% in public variation databases-including the 1,000 Genomes,[3] the Genome Aggregation Database (gnomAD) (v.2.2.1).[4] Variants in the 5' or 3' UTR and variants with intronic locations and no prediction of local splice effect, as well as synonymous variants without prediction of local splice effect were also filtered. Splice effects were predicted using MaxEntScan,[5] NNSplice[6] and SpliceSitFinder.[7] Genes with homozygous and compound heterozygous variants consistent with a recessive mode of inheritance were prioritised for investigation.

Family 2

Three micrograms of genomic DNA were processed according to the SureSelect XT Library Prep protocol (Agilent Technologies, USA). SureSelect Human All Exon V5 (Agilent Technologies) was used as the exome capture reagent. Sequencing was performed using a 100 bp paired-end protocol on an Illumina HiSeq 2500 sequencer (Illumina, USA). The resulting fastq files were aligned to the human reference genome (GRCh37) using Burrows-Wheeler Aligner (BWA, v.0.7.12).[8] The alignment was processed according to GATK best practice. Exome depth was used for CNV analysis according to the developers' guidelines.[9]

Variants were filtered to exclude all changes other than missense, frameshift or stop variants, exonic insertion/deletions or variants located at splice consensus sites. Synonymous variants within the splice consensus region and predicted to affect splicing were also retained. Variants present in the gnomAD (v2.2.1)[4] were excluded if present at a frequency of 1% or higher. Variants were also filtered based on the mode of inheritance. In families known to be consanguineous, homozygous variants were prioritised. Variant lists were also filtered to exclude population specific high frequency variants and platform artefacts by removing variants also present in exomes of individuals of the same ethnicity without dental disease sequenced using the same reagents and platform.

Family 3

One microgram of genomic DNA was processed according to the Agilent SureSelect XT Library Prep protocol (Agilent Technologies). SureSelect Human All Exon V6 (Agilent Technologies) was used as the exome capture reagent. Whole exome libraries were sequenced with a 150 bp paired-end protocol on an Illumina Hi-Seq 3000 sequencer. Alignment, variant calling and filtering were as previously described for Family 2.

Family 4
Investigation of the 100,000 genomes rare disease dataset[10] was carried out using the Gene-Variant Workflow script (available from https://research-help.genomicsengland.co.uk/display/GERE/Gene-Variant+Workflow). This identified all *PLXNB2* variants from all available genome VCF files and annotated them using Ensembl Variant Effect Predictor. A custom Python script called filter_gene_variant_workflow.py (available from https://github.com/sunaynabest/filter_100K_gene_variant_workflow) was used to exclude common variants using the following criteria: 100K major allele frequency (MAF) ≥0.002; gnomAD allele frequency (AF) ≥0.002 and variants called in non-canonical transcripts. Variants were prioritised if they were also marked deleterious by SIFT. Individuals with biallelic high impact variants (variants annotated by VEP as 'high impact' include stop_gained, stop_lost, start_lost, splice_acceptor_variant, splice_donor_variant, frameshift_variant, transcript_ablation, transcript_amplification) were prioritised for study. Variants were excluded if they were only identified in unaffected relatives and not identified in probands. Variant segregation was confirmed by examining the accompanying genome data from parents.

Family 5
50 ng of genomic DNA was processed according to the Human Comprehensive Exome kit (Twist Bioscience) protocol. The exome library was sequenced using an Illumina P3 300 cycle kit on an Illumina NextSeq2000 instrument.

After checking the quality of the raw data by FastQC software (https://www.bioinformatics.babraham.ac.uk/projects/fastqc/) and adaptor trimming by Trim Galore (https://www.bioinformatics.babraham.ac.uk/projects/trim_galore/), sequence alignments were generated against the reference genome (hg19/GRCh37) using the BWA (v0.7.12-r1.39).[8] SAMtools was used to convert SAM files to BAM files and PCR duplicates were removed by Picard (v2.5.0), to produce sorted BAM files.

Variants were filtered to exclude all changes other than missense, frameshift or stop variants, exonic small insertion/deletions or variants located at splice consensus sites. Synonymous variants within the splice consensus region and predicted to affect splicing were also retained. Identified variants were excluded if the CADD (v1.6) score was <15 or minor allele frequency (MAF) was >0.01. The filtered variant list was examined for candidate pathogenic variants in known or potential candidate AI or sensorineural hearing loss genes.

The sorted BAM files were used for custom CNV detection using a pipeline based on the R package ExomeDepth (v1.1.12) using default parameters.[9] A custom reference panel was created using BAM files from another 47 samples sequenced in the same run. Common CNVs[11] were filtered out and CNVs calls prioritised by Bayes factor (the $\log_{10}$ of the likelihood ratio of data for the CNV call divided by that of the normal copy number call).

Soft clipped reads were visualised in Integrated Genomics Viewer to identify CNV breakpoints.

Family 6
Whole exome libraries were prepared from genomic DNA using the SureSelect60Mbv6 (Agilent) reagent using a 100 bp paired-end protocol on a HiSeq 4000 platform (Illumina). Reads were aligned to GRCh37/hg19 using the BWA (v.0.5.87.5).[8] Detection of genetic variation was performed using PINDEL (v 0.2.4t),[12] and ExomeDepth (v 1.0.0).[9] The

cut-off for biallelic inheritance was set to <0.01 allele frequency. The size of reference entries was >27,000 exomes in the in-house database at the time of analysis. Only remaining variants present at <0.01 allele frequency in gnomAD (v2.2.1) were retained.

Missense, nonsense, stoploss, splice, splice consensus, frameshift, and indel variants were included in standard evaluation. Synonymous variants outside of the splice consensus sequence, 5-UTR, 3-UTR, non-coding, miRNA, intronic, intergenic, and regulatory variants were only considered if previously published as pathogenic relevant or if indicated by a distinctive phenotype.[13] For variant interpretation, CADD, Polyphen2, and SIFT scores were used for prioritization. Sanger sequencing for all families was performed with the BigDye Terminator v3.1 kit (Life Technologies) according to the manufacturer's instructions and resolved on an ABI3130xl sequencer (Life Technologies). Results were analysed with SeqScape 2.5 (Life Technologies).

Mouse tissue preparation
Mouse embryos/fetuses were collected at E14.5, E16.5, and on the day of birth (hereafter referred to as E19.5), after matings between C57BL6 mice. For E14.5 and older samples, the whole body was embedded in OCT 4583 medium (Tissue-TEK, Sakura) and frozen on the surface of dry ice. E12.5 embryos were fixed overnight in 4% paraformaldehyde (pH 7.5, w/v) in PBS, cryoprotected by overnight incubation in 20% sucrose (w/v) in PBS, and cryoembedded as described above. Cryosections (Leica CM3050S cryostat) at 10 µm were collected on Superfrost plus slides and stored at –80 °C until hybridization. E14.5 samples were sectioned in a frontal plane, whereas other stages were sectioned sagittally.

Probe Synthesis
The *Plxnb2* probe was synthesized from PCR-generated DNA templates kindly provided by the EURExpress consortium (http://www.eurexpress.org). The template sequence is provided in the supplementary information. DIG-labeled antisense riboprobe was transcribed in vitro by incubation for 2 h at 37 ° C using 1 µg of the PCR product, 20 U SP6 RNA polymerase, 5X transcription buffer (Promega), 10 X DIG RNA labelling mix (Roche), 0.5 M DTT, 20 U RNAse inhibitor (Roche) in a 20 µl volume. The reaction was stopped with 2 µl EDTA (0.2 M, pH 8), and RNA was precipitated with 1 µl yeast tRNA (10 mg/ml), 2.5 µl LiCl (4 M) and 75 µl absolute ethanol, followed by an incubation for 30 min at –80 ° C and centrifugation at 12,000 rpm (30 min at 4° C). The pellet was washed with 70% ethanol and recentrifuged. The supernatant was discarded and the pellet was allowed to dry. The probe was then diluted in 20 µl sterile H_2O. The quality of the probe was verified by electrophoresis in a 1% agarose gel. If no smear was observed and the size was as expected, the probe was considered to be ready for use. The quantity of RNA was evaluated by a Nanodrop spectrophotometer (ND-1000, Labtech) and adjusted to 150 ng/ µl in hybridization buffer, then stored at –20 °C until use.

In situ Hybridization
Slides were allowed to thaw to room temperature (RT) for 2 h. Then they were post-fixed on ice in 4% paraformaldehyde (diluted in PBS) for 10 min and rinsed in PBS. The hybridization buffer was composed of 50% deionized formamide, 10% dextran sulfate, 1mg/ml yeast tRNA, 1 X Denhardt's solution, and 1 X salt solution (0.195 M NaCl, 5 mM Tris pH 7.2, 5 mM $NaH_2PO_4.H_2O$, 5 mM $Na_2HPO_4.12H_2O$, 5 mM EDTA pH 8). The probe was diluted in hybridization buffer at a concentration of 1 µg/ml. The probe mix was denatured by a 10 min incubation at 70 °C and placed on ice. 100 µl was applied on each slide and covered by a coverslip. This was allowed to hybridize overnight at 65 °C in

humidified chambers. The slides were then washed twice for 30 min at 65 °C in 1X standard saline citrate (SSC), 50% formamide, 0.1% Tween-20, and twice for 30 min at RT in MABT buffer (1 X MAB (maleic acid buffer): 0.5 M maleic acid (Roche), 0.75 M NaCl pH 7.5, 0.1% Tween-20). Probe detection was performed using antibodies and reagents from Roche. Slides were incubated for 1 hr at RT with a blocking solution (20% goat serum, 2% blocking reagent in MABT). The anti-DIG antibody was diluted 1: 2,500 in blocking solution, and 200 µl was added to each slide, which were covered by Parafilm and incubated overnight at 4 ° C. Slides were washed 5 times in MABT for 20 min and then twice for 10 min in NTMT buffer (100 mM NaCl, 100 mM Tris-HCl pH 9.5, 50 mM $MgCl_2.6H_2O$, 0.1% Tween-20). An aliquot of 200 µl of freshly prepared staining solution (3.5 µl nitro-blue tetrazolium chloride (Roche), 3.5 µl 5-bromo-4-chloro-3'indolylphosphate p-toluidine salt (Roche) in NTMT buffer) was placed on each slide, covered by a Parafilm and incubated overnight in the dark at RT. The staining solution was changed every day and when signal was optimal, the slides were rinsed twice for 5 min in NTMT buffer. The slides were further rinsed with PBS and water, allowed to dry overnight, and mounted in Coverquick 2000 mounting medium (Labonord).

**Target (GRCh37)**	**Forward primer (5¢)**	**Reverse primer (3¢)**	**Target (bp)**	**Primer Identifier (Use)**
chr22:50722118-50722591	TACGGCAAGAATATCGACAGCAAG	AAATTGGACCCCAGGATGGTGA	474	Exon 14 (Family 1)
chr22:25600810-25601544	TGGGAAGATTAAACCGGCATCTGG	TGAGGCAGGAGAATGGCATGAAC	735	*CRYBB3* exon 5 (Family 1)
chr22:50722428-50722719	GAGGCTGCCCTTGTGACC	AGAGCTGCCGTCAGTGGT	292	Exon 13 (Family 2)
chr22:50721764-50722004	ACTTGGAAGGTGAACTGGACA	CTGTCAGCTGGGTCAGTACC	241	Exon 16 (Family 4)
chr22:50719090-50719291	TGTGGATGAACTGTAGGGGC	GCGACAAGGACGTGATGATC	202	Exon 24 (Family 4)
chr22:50715038-50715798	GTCCCTGGTGGTCTCCTTAC	CAGCACACGAGCAGAGAAAC	761	F5-R7 (Family 5)
chr22:50714866- 50715798	AAGCAGGAGCAGCTCATACT	CAGCACACGAGCAGAGAAAC	933	F6-R7 (Family 5)
chr22:50716913-50717351	GATCATTGACCAGGTGTACCG	CACCTTGGACAGGATGAGG	439	Exon 29 (Family 6)

Supplementary Table 1. Primers used to verify the variants identified in Families 1, 2, 4-6. Primers are for regions of *PLXNB2* unless otherwise stated. Coordinates are for the GRCh37 human reference genome.

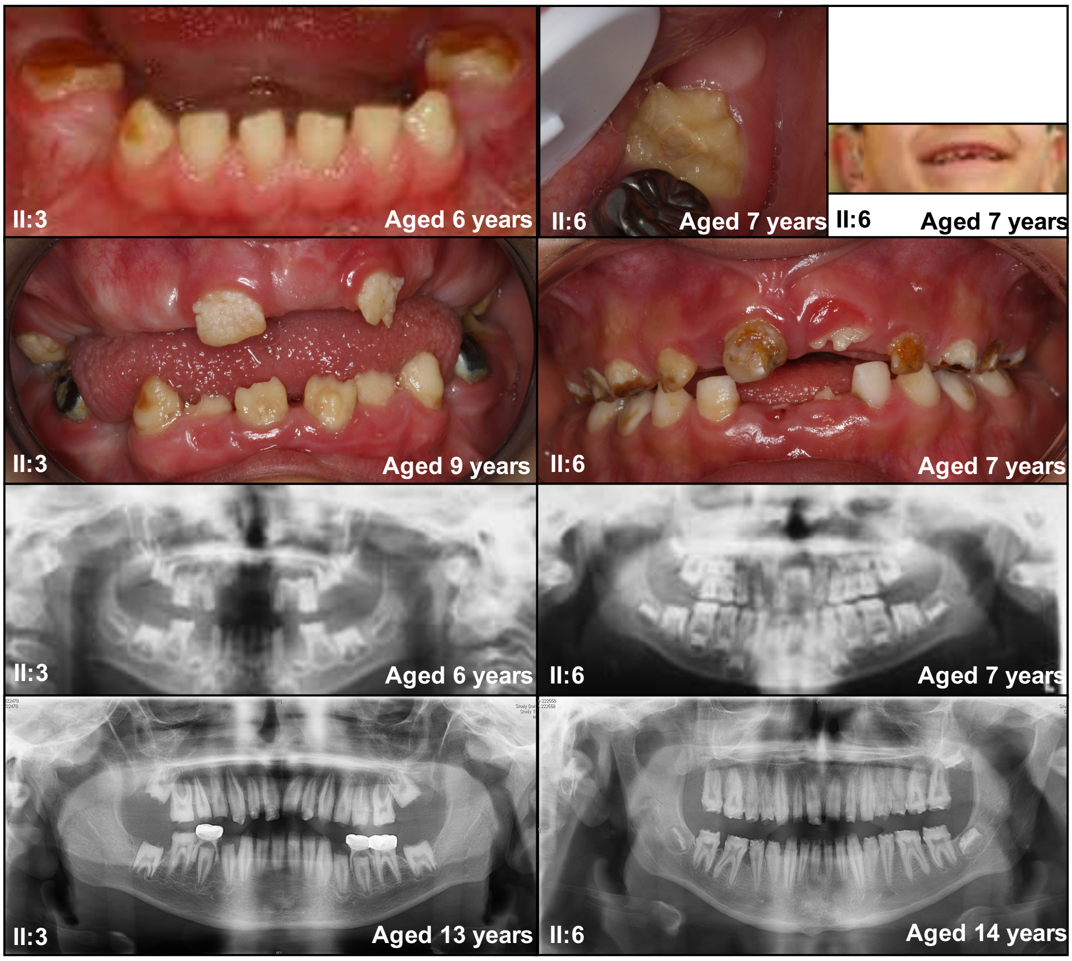

Supplementary Figure 1. Family 1: Clinical images of affected individuals. Oral photographs and panoramic radiographs are shown for II:3 and II:6 which illustrate changes over time. The dentitions of both affected individuals are characterised by variable degrees of enamel hypoplasia AI with only a thin layer of enamel present in some places. Crown morphology is atypical in some teeth with striking flattening of normal cusp morphology of the permanent molar teeth evident on radiographs prior to eruption. The radiographic appearances of the dental pulp spaces are within expected limits other than mild taurodontism in some permanent molar teeth. The root morphology is within expected limits. Individual II:6 is shown with bilateral hearing aids.

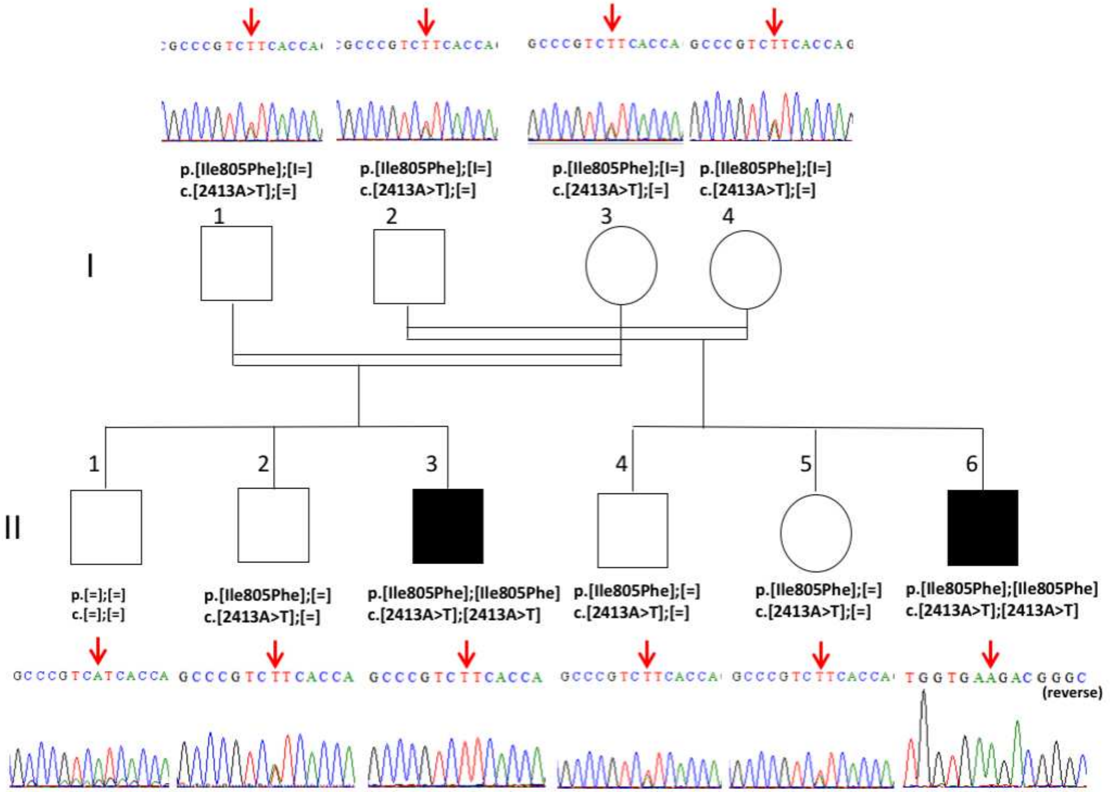

Supplementary Figure 2. Segregation of the *PLXNB2* variant c.2413A>T, p.(Ile805Phe) identified by exome sequencing of II:3 and II:6.

Family carrying variant and allelic status	Variant (GRCh37)	Transcript (Exon) ENST00000359337.9 / NM_012401.4	Protein	CADD v1.6 GRCh37	REVEL	Polyphen -2 (Hum Var)	SIFT	gnomAD v2.1.1; dbSNP155
1 homozygous	g.50722270T>A	c.2413A>T 14	p.(Ile805Phe)	25.2	0.649	0.97 predicted damaging	0 deleterious	Not present in either
2 homozygous	g.50722576C>T	c.2248G>A 13	p.(Asp750Asn)	23.6	0.159	0.177 benign	0.01 deleterious	Not present; rs1332617247
3 compound heterozygous	g.50728264G>T	c.750C>A 3	p.(Cys250*)	36	N/A	N/A	N/A	Not present in either
3 compound heterozygous	g.50720613C>T	c.3117G>A 19	p.(Thr1039=)	22.7	N/A	N/A	N/A	Not present; rs1234372437
4 compound heterozygous	g.50721841delA	c.2606del 16	p.(Phe869Serfs*45)	17.61	N/A	N/A	N/A	4.024×10^{-6} 1/248518; not present
4 compound heterozygous	g.50719182_50719186 delAAAG	c.3982_3986del 24	p.(Phe1328Hisfs*65)	34	N/A	N/A	N/A	Not present in either
5 homozygous	g.50715085_50715672 del	c.5197-337_5310del 34-35	p.(Asp1733_Arg1779del)♦	N/A	N/A	N/A	N/A	N/A
6 homozygous	g.50717063C>T	c.4609G>A 29	p.(Gly1537Ser)	29.1	0.297	0.973 probably damaging	0 deleterious	Not present in either

Supplementary Table 2. Variant interpretation and population frequency data of the *PLXNB2* variants identified in the individuals included in this study. Various pathogenicity prediction scores are included to aid variant interpretation, including Combined Analysis Dependent Depletion (CADD),[14] REVEL,[15] Polyphen-2[16] and SIFT.[17] The population frequency of each variant in the gnomAD database[4] and its presence or absence in dbSNP155 is also shown. The Genomics England GRCh37 cohort was also checked for these variants, but none were present. Analysis by Franklin (https://franklin.genoox.com), showed all variants were classified as VUS (data not shown). Variants are based on genome build GRCh37 and *PLXNB2* transcript ENST00000359337.9, NM_012401.4 and PLXNB2 protein ENSP00000352288.4, NP_036533.2. ♦Note that splice prediction software predicts the skipping of all of exon 35, despite only partial deletion.

```
                             Asp750
Zebrafish        NVTFYIKDKDTDRKIDSTLRVVLYNCSVRREDCSLCKNAHKNDSCVWCDT---TKSCIYR 818
Chicken          KLKLSMR--TGKVKIDSKLMVTLYNCSYGRSDCSLCLAAHEDYKCVWCEEDGKPSKCVYE 804
Mouse            PLHLYVK--SFGKNIDSKLQVTLYNCSFGRSDCSLCLAADPAYRCVWCRG---QNRCVYE 793
Human            PLHLYVK--SYGKNIDSKLHVTLYNCSFGRSDCSLCRAANPDYRCAWCGG---QSRCVYE 791
Gorilla          PLHLYVK--SYGKNIDSKLHVTLYNCSFGRSDCSLCRAANPDYRCAWCGG---QSRCVYE 791
Red deer         PLHLYVK--SSGKNVDSRLQVTLYNCSFGRSDCSLCLAADPAYRCVWCSG---QSRCVYE 795
Dog              PLHLYVK--SYGKNVDSKLQVTLYNCSFGRSDCSLCLAADPAYKCVWCAG---QGRCVYE 793
Cat              PLHLYVK--SYGKNVDSKLQVTLYNCSFGRSDCSLCLAADPVYKCVWCGG---QSRCVYE 793
Giant panda      PLHLYVK--SYDKNVDSKLQVTLYNCSFGRSDCSLCLAADPAYKCVWCAG---QSGCVYE 793
Pig              PLHLYVK--SYGKNIDSRLQVTLYNCSFGRSDCSLCLAADPAYKCVWCSG---QSRCVYE 793
Narwhal          PLHLYVK--SYGKNIDSRLQVTLYNCSFGRSDCSLCLAADPVYRCVWCSG---QSRCVYA 793
Baleen whale     PLHLYVK--SYGKNIDSRLQVTLYNCSFGRSDCSLCLAADPAYQCVWCSG---QSGCVYA 793
                  : : ::      ::** * *.*****  *.*****  *.    *.**        *:*

                             Ile805
Zebrafish        SLCDNE-LHQCPPPKITDIVPRYGPMNGRISVTIKGSNLGIKKEDIKKITVAKVPCIHQP 877
Chicken          KLCRAPPTNTCPHPEITRIEPKTGPLDGGILLTIMGSNLGIKAEDVKNITVADKECVFKE 864
Mouse            ALCSNV-TSECPPPVITRIQPETGPLGGGILVTIHGSNLGVKADDVKKITVAGQNCAFEP 852
Human            ALCN-T-TSECPPPVITRIQPETGPLGGGIRITILGSNLGVQAGDIQRISVAGRNCSFQP 849
Gorilla          ALCN-T-TSECPPPVITRIQPETGPLGGGIRITILGSNLGVQAGDIQRISVAGRNCSFQP 849
Red deer         ALCSNA-TSECPPPVVTRIQPETGPLGGGIRITILGSNLGVRADDVKRVTVAGQNCAFEP 854
Dog              ALCSNT-TSECPPPVITRIHPETGPLGGGIRITILGSNLGVTADDVKRVTVAGQNCAFEP 852
Cat              ALCSNA-TSECPPPVITRIHPETGPLGGGIRITILGSNLGVRADDVKRITVAEQNCVFQP 852
Giant panda      ALCSNT-TSECPPPVITRIHPETGPLGGGIRITIVGSNLGVRAEDVRGVTVAGQNCVFEP 852
Pig              ALCSNA-TSECPPPVITRIHPETGPLGGGIHVTILGSNLGVKADDVTRVTVAGRNCTFEP 852
Narwhal          ALCGNA-TSECPPPVITRIQPETGPLGGGIRVTILGSNLGVRADDVKRVTVAGRSCAFEP 852
Baleen whale     ALCGHA-TSECPPPVITRIQPETGPLGGGIRITILGSDLGVRADDVKRVTVAGQNCAFEP 852
                  **       ** * :* * *. **:.* * :** **:**:   *:  ::**   * .:
```

```
                                                         Gly1537
Zebrafish      IEQVYRNLPYSQRPVVDSVALEWRPGSTGQILSDLDLTSQKEGRWKRINTLAHYNVRDGA 1581
Chicken        IDQVYRNLPCSQWPKAESVALEWRPGSTAQILSDLDLTSQRDGRWKRINTLMHYNVRDGA 1561
Mouse          IDQVYRTQPCSCWPKPDSVVLEWRPGSTAQILSDLDLTSQREGRWKRINTLMHYNVRDGA 1558
Human          IDQVYRGQPCSCWPRPDSVVLEWRPGSTAQILSDLDLTSQREGRWKRVNTLMHYNVRDGA 1554
Gorilla        IDQVYRGQPCSCWPRPDSVVLEWRPGSTAQILSDLDLTSQREGRWKRVNTLMHYNVRDGA 1554
Red Deer       IDQVYRTQPCSRWPKADSVVLEWRPGSTAQILSDLDLTSQREGRWRRVNTLMHYNVRDGA 1559
Dog            IDQVYRTQPCSRWPKADSVVLEWRPGSTAQILSDLDLTSQREGRWKRINTLMHYNVRDGA 1557
Cat            IDQVYRTQPCSRWPKADSVVLEWRPGSTAQILSDLDLTSQREGRWKRVNTLMHYNVRDGA 1557
Giant panda    IDQVYRTQPCSRWPKADSVVLEWRPGSTAQILSDLDLTSQREGRWKRINTLMHYNVRDGA 1557
Pig            IDQVYRTQPCSRWPKADSVVLEWRPGSTAQILSDLDLTSQREGRWKRFNTLMHYNVRDGA 1557
Narwhal        IDQVYRTQPCSRWPKADSVVLEWRPGSTAQILSDLDLTSQREGRWKRVNTLMHYNVRDGA 1556
Baleen whale   IDQVYRTQPCSRWPKADSVVLEWRPGSTAQILSDLDLTSQREGRWKRVNTLMHYNVRDGA 1557
               *:****  * *  *  :**.********.***********::***:*.*** ********
```

Supplementary Figure 3. Multiple sequence alignment of PLXNB2 sequences from a variety of species to show the evolutionary conservation of the mutated residues suggested to be disease causing. The evolutionary conservation for residues Asp750 (Family 2), Ile805 (Family 1) and Gly1537 (Family 6) and surrounding residues is shown. Disease causing residues are highlighted by a red box. Human (*Homo sapiens*) NP_001363798.1; mouse (*Mus musculus*); gorilla (*Gorilla gorilla gorilla*) XP_030863195.1; pig (*Sus scrofa*) XP_020946968.1; dog (*Canis lupus familiaris*) XP_038535376.1; cat (*Felis catus*) XP_044918511.1; red deer (*Cervus elaphus*) XP_043737305.1; giant panda (*Ailuropoda melanoleuca*) XP_034499687.1; chicken (*Gallus gallus*) XP_046764781.1; narwhal (*Monodon monoceros*) XP_029061558.1; baleen whales (*Balaenoptera musculus*) XP_036721225.1; zebrafish (*Danio rerio*) NP_001155072.1.

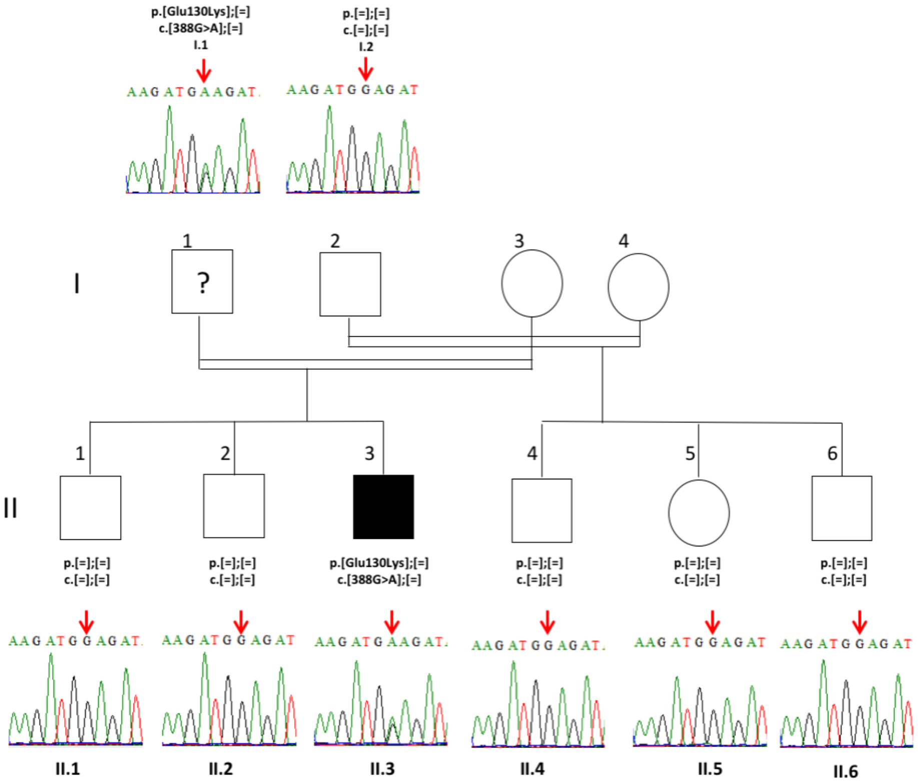

Supplementary Figure 4. Segregation of the *CRYBB3* variant c.388G>A, p.(Glu130Lys) identified by exome sequencing of II:3. The variant was inherited from his father (I:1). It is unknown whether I:1 has cataracts.

**Family**	**Other genetic findings**	**Reference sequences**
1	chr22:25601247G>A, Homozygous *CRYBB3* variant c.388G>A, p.(Glu130Lys). Did not segregate with disease for all family members, but was likely responsible for cataract phenotype in some individuals (Supplementary Figure 4).	MANE Select transcript NM_004076.5 (ENST00000215855.7) RefSeq protein NP_004067.1 (ENSP00000215855.2)
2	Chr21:47544826G>A, Homozygous *COL6A2* variant c.1762G>A:p.(Gly588Ser) [3 hets in gnomAD] segregated with disease for available family members CADDv1.6 score 27.1, in dbSNP (rs139488626), in ClinVar as a benign variant / variant of uncertain significance. However, the published COL6A2 disease phenotype is myopathy which was not observed in the affected individual examined. Homozygous variants with CADD>15 were also observed in *AQP1*/*FAM188B* (one variant in multiple transcripts) and *TRAK1*, but variants in these genes have no relevant known disease association and the encoded proteins have no obvious link with symptoms observed in patients, so they were not investigated further.	MANE Select transcript NM_0041849.4 (ENST00000300527.9) RefSeq protein NP_001840.4 (ENSP00000300527.4)
3	No other relevant variants identified.<br>Known and candidate retinal disease genes were specifically checked as this individual was enrolled into a study on inherited retinal disease. The *PLXNB2* variant was scored highest by pathogenicity prediction software after variant filtering, with no other variants with comparable scores.	
4	Genomes England data had undergone tiering. No other likely causative variants for the phenotypes that the individual was entered into the study under were apparent in Tiers 1-3. The top hit variants for Exomiser were compound heterozygous variants in *FLNB* (comp het, c.2773G>T, p.(Gly925Cys) [0.1% in gnomAD] and c.124C>T, p.(Arg42*) [absent in gnomAD]). The only other known disease gene with biallelic variants was *PKD1* (comp het, c.11800G>A, p.(Gly3934Arg) [1 het in gnomAD] and c.872C>T, p.(Ser291Leu) [absent from gnomAD but only present in ENST00000567946, not the MANE Select one]). For both genes, their reported disease phenotype does not fit with the disease phenotype of the affected individual.	*FLNB* MANE Select transcript NM_001457.4 (ENST00000295956.9) FLNB RefSeq protein NP_001448.2 (ENSP00000295956.5)<br><br>*PKD1* MANE Select transcript NM_001009944.3 (ENST00000262304.9) PKD1 RefSeq protein NP_001009944.3 (ENSP00000262304.4)
5	Family 5 was identified as having a similar phenotype by the same clinician treating family 4. Homozygous variants with CADD>15 were identified in *DENNDH6B*, *RHBDL1*, *TSC2*, *RIMS1*, *GPBP1*, *PKD1*, *SMPD3* and *COX3*. No other rare variants in genes for which variants are known to cause aspects of the phenotype observed in this patient or in candidate genes were identified. The phenotypes expected for these genes, where already known, either do not match the mode of disease inheritance or the phenotype of the affected individual. Parents were noted to be unaffected by disease.	N/A

Family	Other genetic findings	Reference sequences
6	Family 6 was identified through Genematcher, where labs report variants in genes believed to be causative for disease but for which only have one family identified. This flagged an individual with biallelic *PLXNB2* variants with an overlapping disease phenotype to the other cases. The homozygous variant identified in *PLXNB2* was scored highest by pathogenicity prediction software after variant filtering, with no other homozygous variants with comparable scores.	N/A

Supplementary Table 3. Details of other genetic findings in affected individuals after variant filtering.

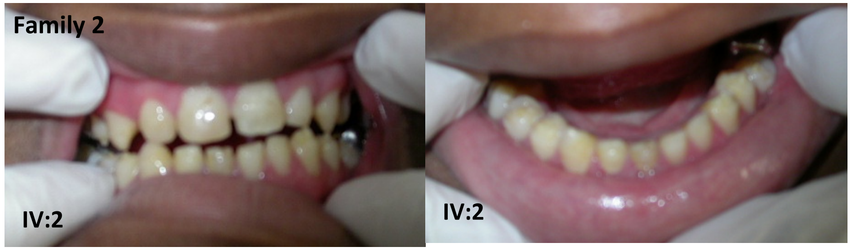

Supplementary Figure 5. Family 2: Clinical images of IV:2 are consistent with hypomineralised AI.

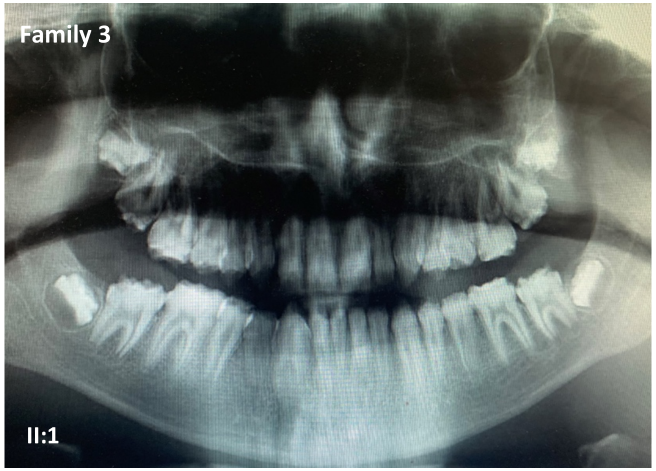

Supplementary Figure 6. Family 3: **II:1** The dental panoramic tomograph (OPT) captures the abnormal dental enamel formation with variable enamel hypoplasia and crown morphology that is especially evident in the unerupted teeth (arrowhead) where the cervical enamel is a more normal morphology compared to the occlusal enamel, which is markedly hypoplastic. These observations are consistent with those for the OPT presented for Family 4 II:1.

Genotype	pos 5'->3' phase strand	confidence	5' exon intron 3'
Wildtype	721 0 +	0.82	GCCCATGACG^GTCAGTCCTG
Mutant	ND		

Supplementary Table 4. NetGene2 (v2.4.2) donor splice site predictions for the effect of the variant identified in Family 2. A section spanning the flanking introns and exons was analysed (chr22:50720258-50721332, based on genome build GRCh37, reverse complement), selected results are shown. Wildtype and mutant *PLXNB2* (g.50720613C>T) were investigated. ND not detected.

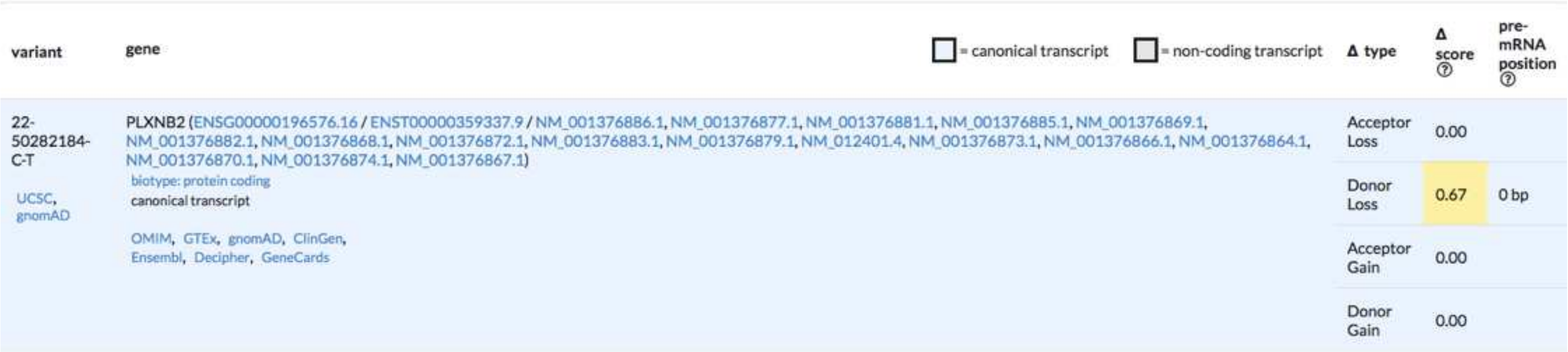

Supplementary Figure 7. SpliceAI predictions for the effect of the variant identified in Family 2. SpliceAI gave the variant a delta score of 0.67 for splice donor loss, which is above the threshold for alterations of splicing of 0.5.

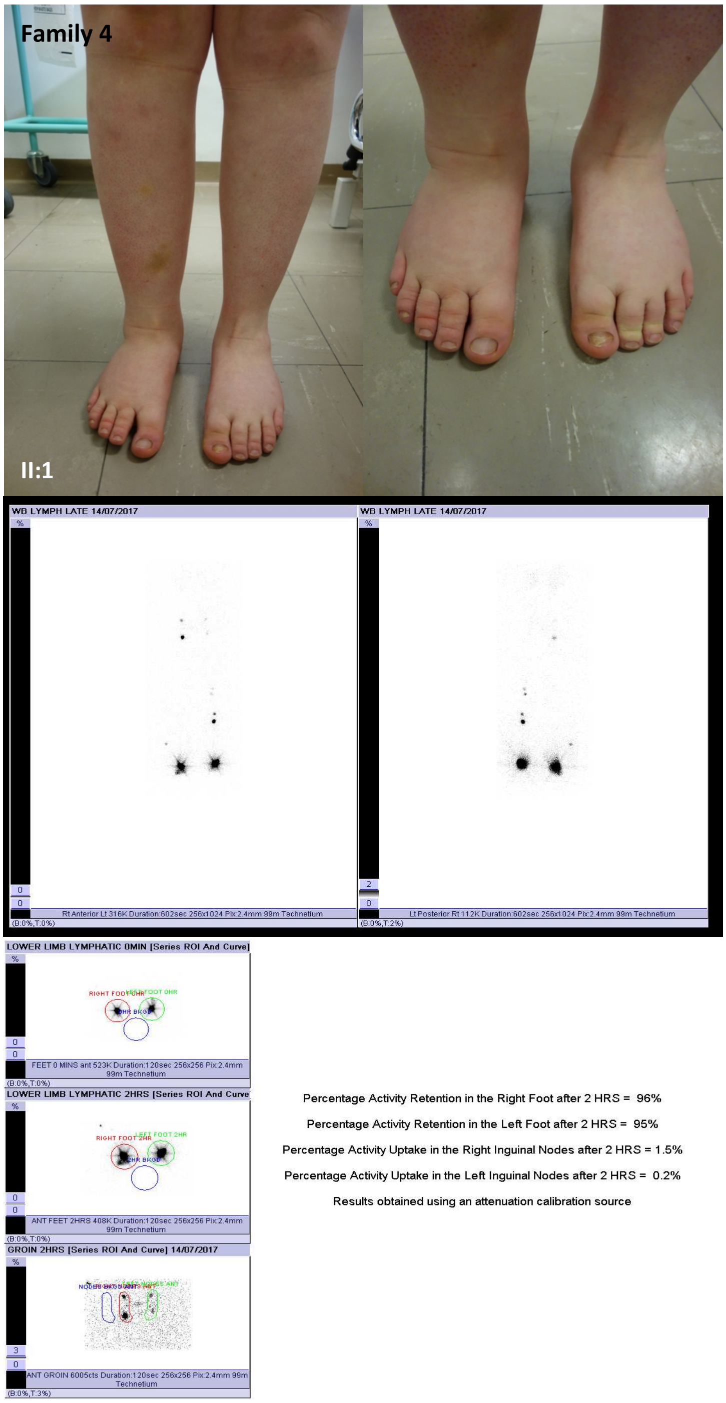

Supplementary Figure 8. Family 4: Bilateral lower limb lymphoedema in Family 4 II:1. Images show bilateral lower limb lymphoedema. Bilateral lower limb lymphoscintigraphy imaging at 2 hours after injecting radioactive ^{99}technetium into toe web spaces is also shown. Imaging demonstrates poor uptake into the inguinal lymph nodes with few nodes visualised. The uptake into the inguinal lymph nodes at 2 hours was 1.5% on the right and 0.2% on the left, in contrast, normal uptake is >12%. The appearance of popliteal lymph nodes on the left suggesting deep rerouting of the lymphatic tracts.

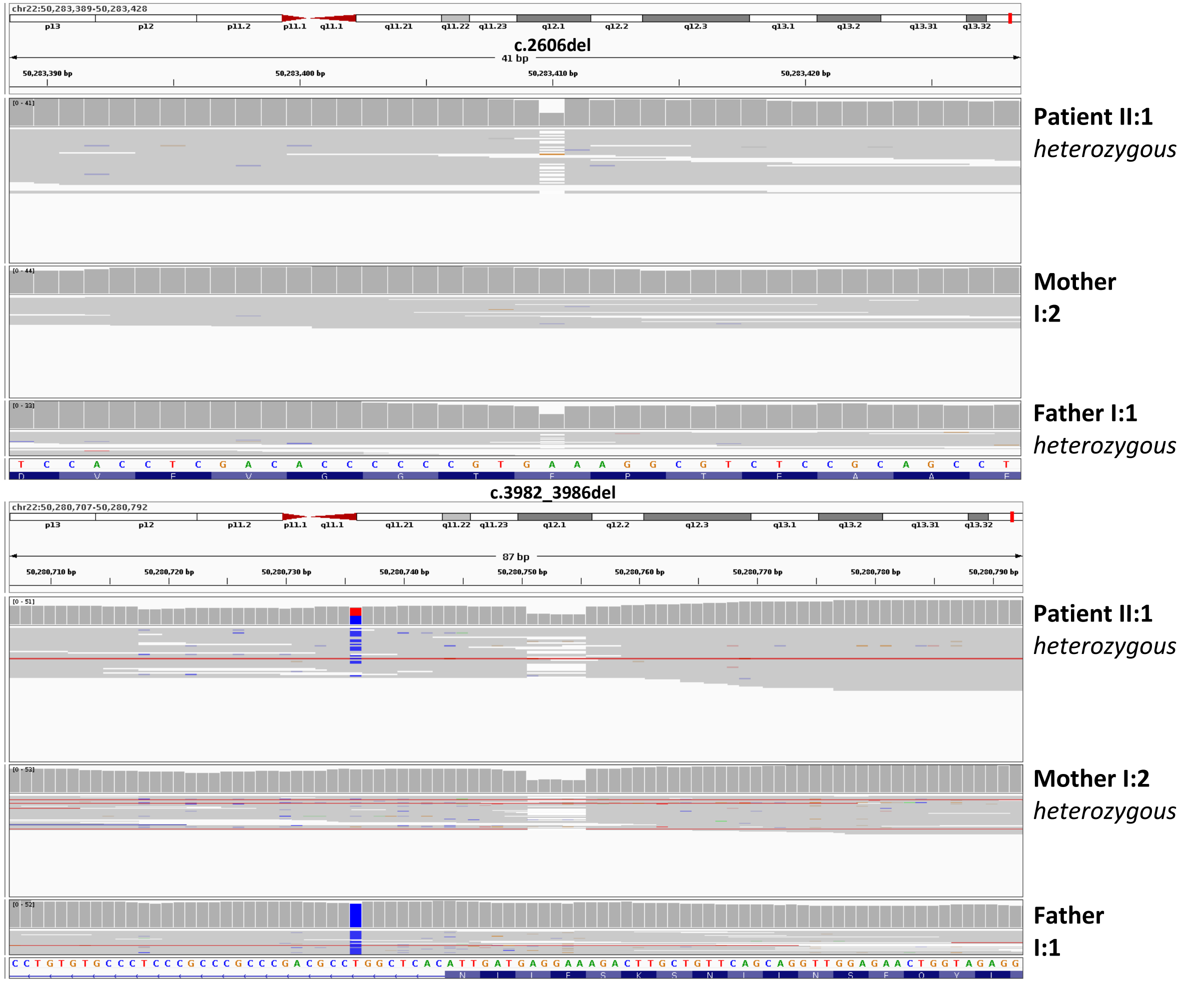

Supplementary Figure 9. IGV images of the variants positions (GRCh38) for individuals from Family 4. IGV traces show c.2606del (upper panel) and c.3982_3986del (lower panel) showing the reduction in read depth relative to the surrounding positions and in comparison to the sequencing of other family members.

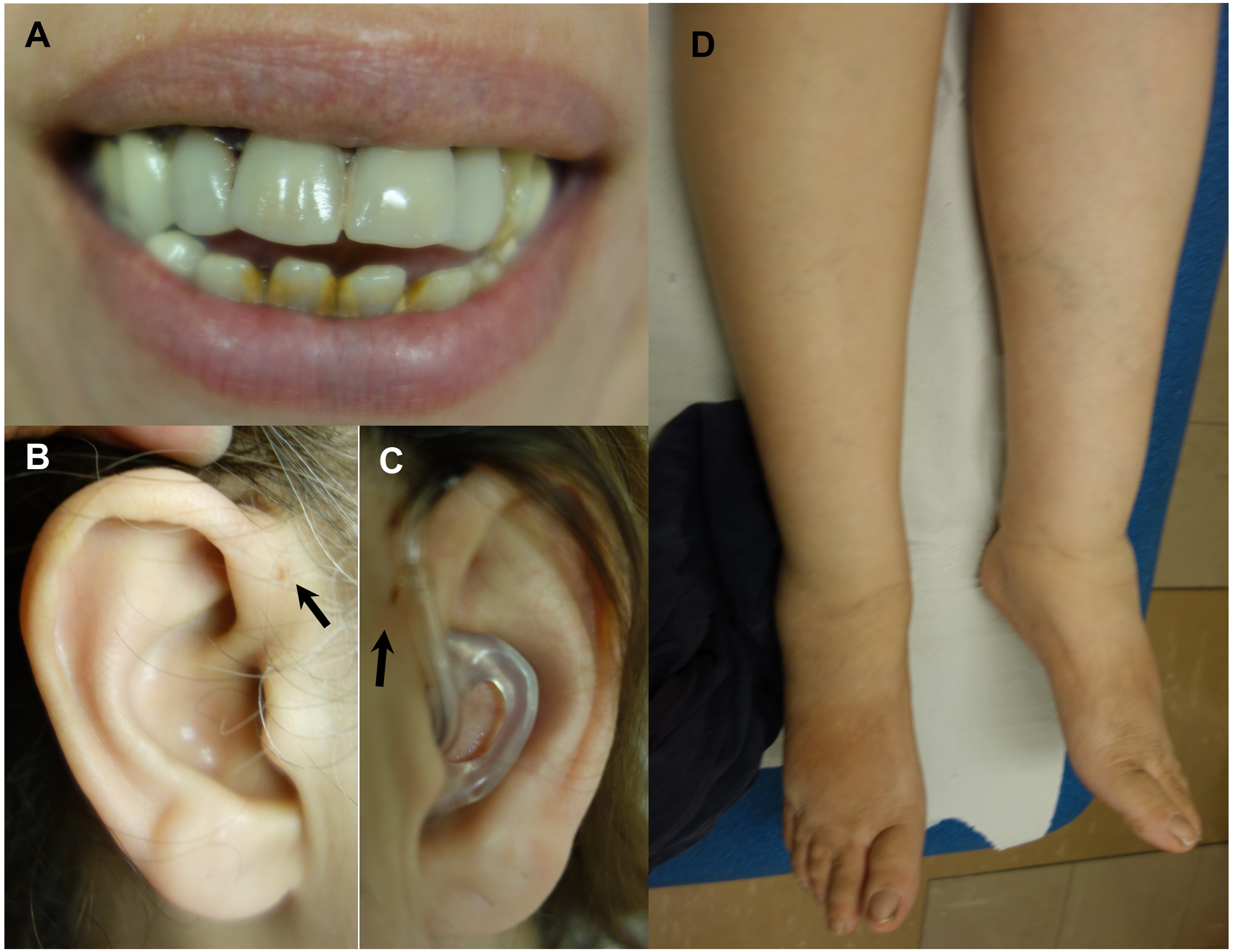

Supplementary Figure 10. Clinical images of Family 5 II:1. A The permanent dentition has been extensively restored and there are calculus deposits on the lower anterior teeth. B and C Periauricular pits are present (blind ended fistulas; arrows). The individual uses a hearing aid. D Bilateral lymphoedema of the lower limbs.

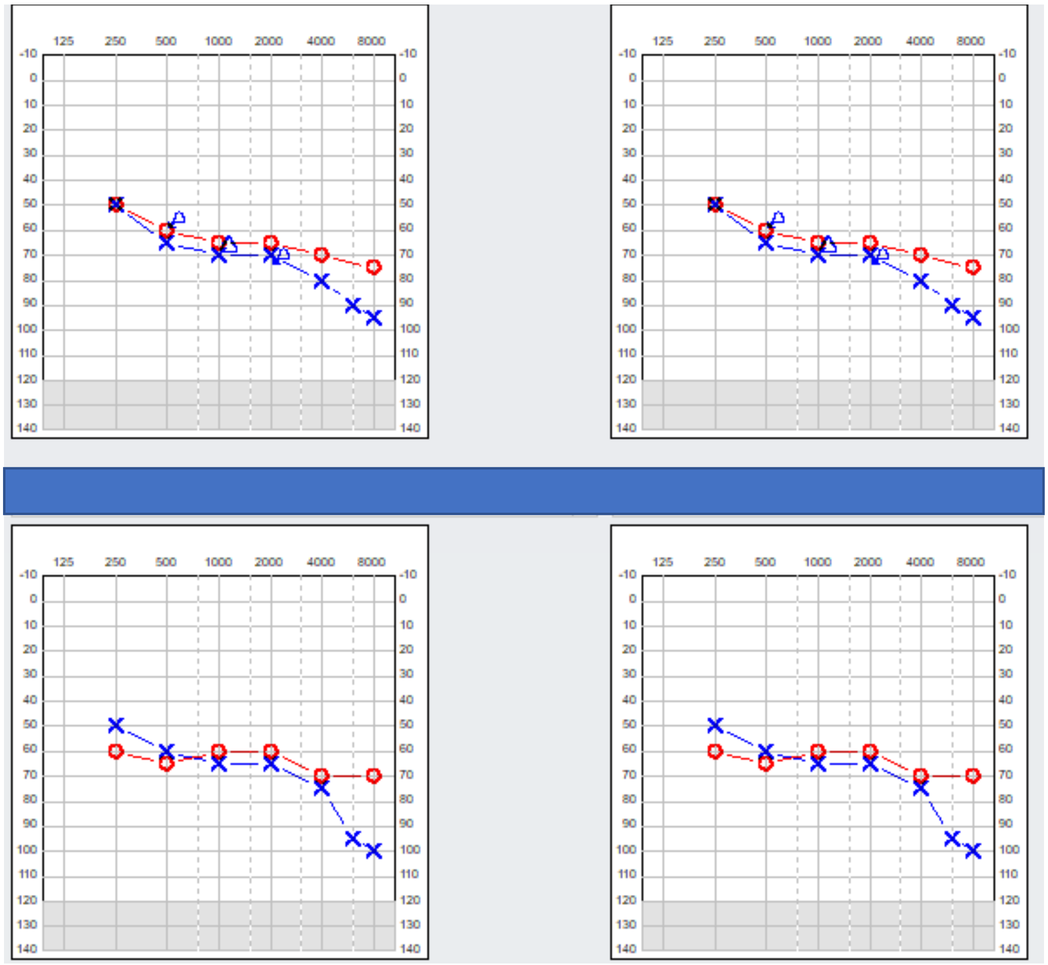

Supplementary Figure 11. Auditory exam history of Family 5 II:1. Results for the left ear (blue) and right ear (red) with headphones are shown for sound frequencies 250-8000 Hz. Results for tests carried out in the past year (top left), one year earlier (top right) and five years earlier are included (both tests shown at the bottom).

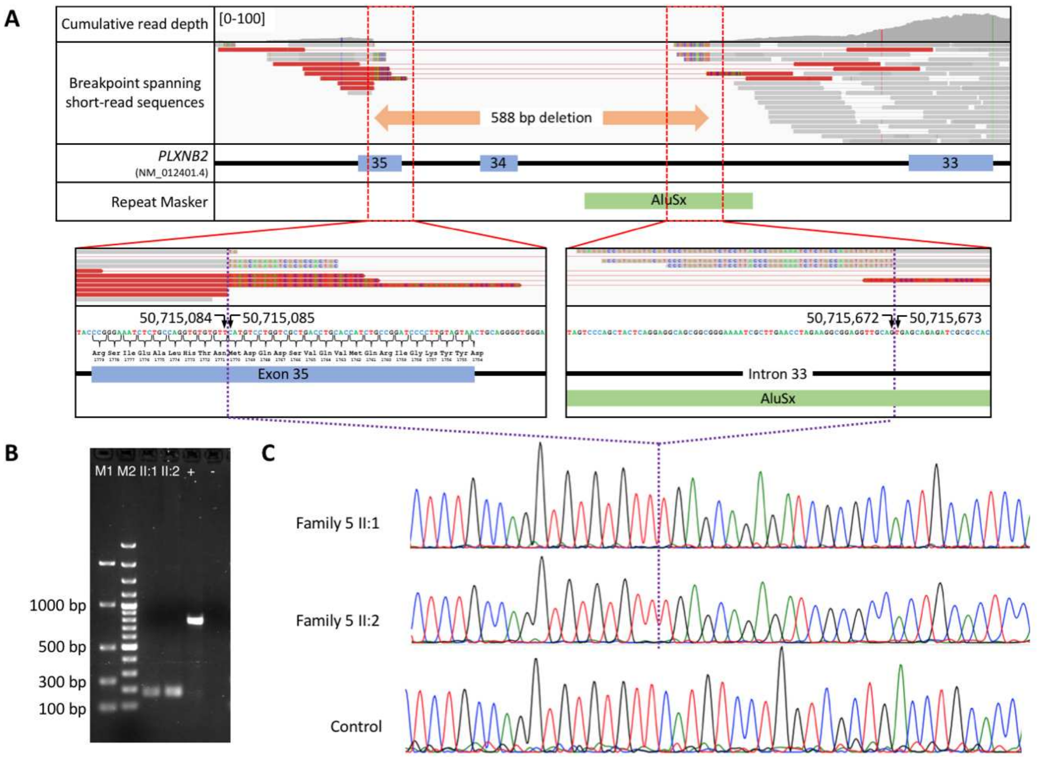

Supplementary Figure 12. Characterisation of the *PLXNB2* deletion in Family 5 by whole exome and Sanger sequencing. A Diagram showing the results of short read data analysis. Soft clipped reads revealed the breakpoints to be at chr22:50715085 and chr22:50715672 (based on genome build GRCh37), resulting in the complete deletion of exon 34 and a partial deletion of exon 35, c.5197-337_5310del (NM_012401.4), predicted to result in an in-frame deletion p.(Asp1733_Met1770del) (NP_036533.2), however splicing prediction predicts that the remainder of exon 35 will be skipped, resulting in p.(Asp1733_Arg1779del). B Gel image showing products resulting from PCR amplification using primers F5-R7 (Supplementary Table 1). DNA from both II:1 and II:2 but not a control individual produces an approx. 175 bp product when a product of 760 bp is expected. Legend: M1: EasyLadderI (Bioline), M2: 100 bp ladder (Fermentas), + control DNA, - water. C Sanger sequencing, using F6-R7 (Supplemental Table 1). confirmed these breakpoint coordinates, revealed that the same deletion was also present in the affected sibling and showed that there were no other alterations around the breakpoints.

Genotype	pos 5'->3' phase strand	confidence	5' exon intron 3'
Wildtype	158 2 +	0.95	TCTGCCTCAG^CTTACCGCTC
Mutant	ND		

Supplementary Table 5. NetGene2 (v2.4.2) acceptor splice site predictions for the effect of the deletion identified in Family 5. A section spanning the flanking introns and exons was analysed (chr22:50,713,408-50,716,325, reverse complement), selected results are shown. Wildtype and mutant *PLXNB2* (g.50715085_50715672del) were investigated. No alternative splice acceptor site was detected. ND not detected.

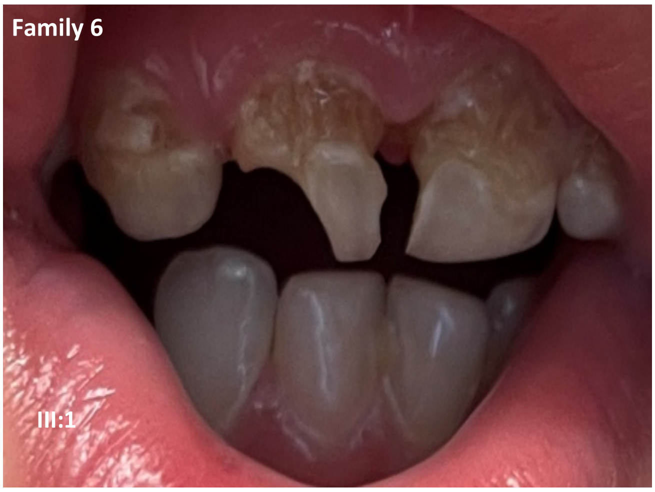

Supplementary Figure 13. Family 6: Examination of the dentition of III:1 was limited by patient compliance, but extensive post-eruptive enamel changes were clear, with dental caries as a contributory factor.

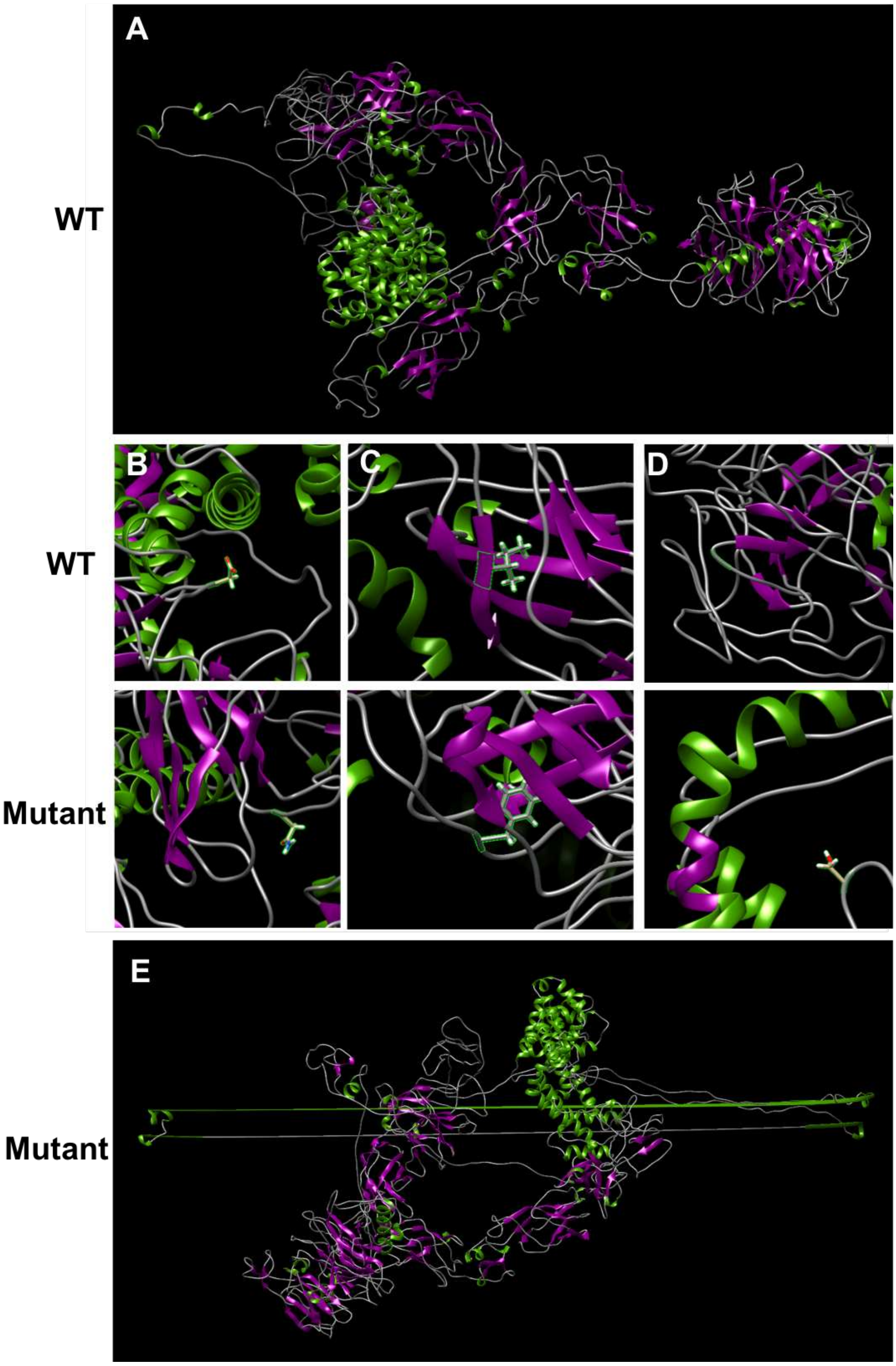

Supplementary Figure 14. Predicted tertiary structure of PLXNB2 using the I-Tasser-MTD algorithm. A Wild-type (WT) protein structure. B Comparison of WT with Asp750Asn mutant structure. C Comparison of WT with Ile805Phe mutant structure. D Comparison of WT with Gly1537Ser mutant structure. E. Mutant p.(Asp1733_Arg1779del) protein structure.

CAAAGCAGATGACGTCAAGAAGATAACTGTGGCTGGCCAGAACTGTGCCTTTGA
ACCAAGAGGGTACTCCGTATCCACCCGGATTGTGTGTGCAATTGAGGCTTCGGAG
ATGCCCTTCACAGGAGGCATTGAGGTGGATGTTAATGGAAAACTCGGCCATTCA
CCGCCACACGTCCAGTTCACTTATCAACAACCCCAGCCTCTCAGTGTGGAGCCAC
GACAGGGGCCACAGGCAGGTGGCACCACATTGACCATCAATGGCACTCACCTGG
ACACAGGCTCCAAGGAGGATGTGCGGGTGACACTCAATGACGTCCCTTGTGAAG
TGACAAAGTTTGGAGCACAGCTACAGTGTGTCACAGGTCAACAGTTGGCTCCAG
GCCAGGTGACACTAGAAATCTACTATGGGGGCTCCAGAGTGCCCAGCCCCGGCA
TCTCTTTCACCTACTGCGAGAACCCCATGATACGAGCCTTTGAGCCATTGAGAAG
CTTTGTCAGTGGTGGCCGGAGCATCAACGTTACTGGCCAGGGCTTCAGCCTCATC
CAGAAGTTTGCCATGGTTGTCATCGCTGAGCCCTTGCGGTCCTGGAGGCGGCGGC
GGCGGGAGGCTGGAGCCCTGGAGCGTGTGACGGTCGAGGGCATGGAGTACGTGT
TCTACAACGACACCAAGGTCGTCTTCTTGTCTCCTGCTGTCCCCGAAGAGCCCGA
GGCTTACAACCTCACCGTGCTGATA

Supplementary Figure 15. Murine *Plxnb2* template sequence (733 bp) used for in situ hybridization.

BMJ Publishing Group Limited (BMJ) disclaims all liability and responsibility arising from any reliance placed on this supplemental material which has been supplied by the author(s)

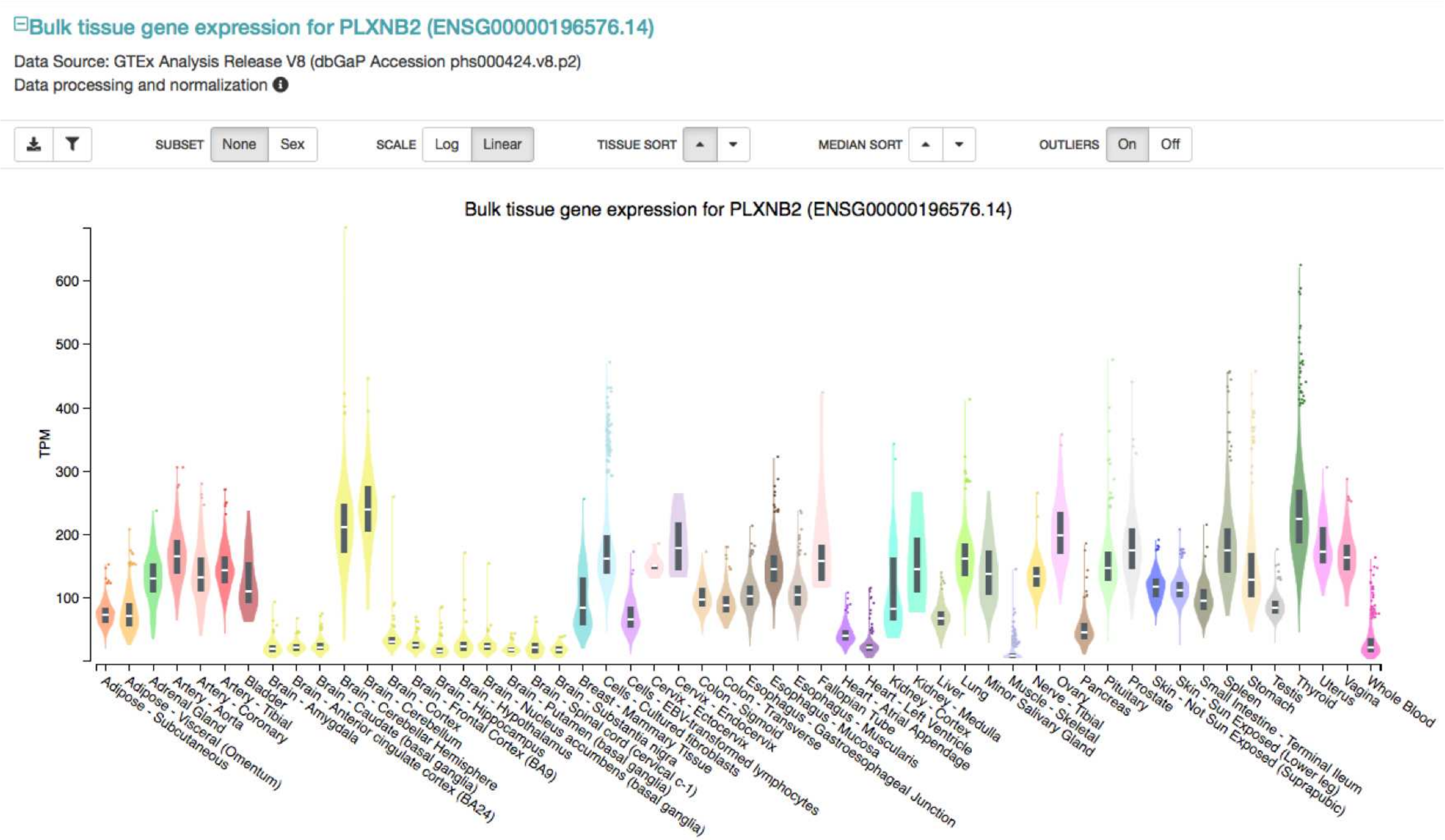

Supplementary Figure 16. Bulk tissue gene expression for *PLXNB2* from GTEx portal.[18]

References

1 Laugel-Haushalter V, Bär S, Schaefer E*, et al.* A New SLC10A7 Homozygous Missense Mutation Responsible for a Milder Phenotype of Skeletal Dysplasia With Amelogenesis Imperfecta. *Front Genet* 2019;10:504. doi:10.3389/fgene.2019.00504

2 Backenroth D, Homsy J, Murillo LR*, et al.* CANOES: detecting rare copy number variants from whole exome sequencing data. *Nucleic Acids Res* 2014;42:e97. doi:10.1093/nar/gku345

3 Abecasis GR, Auton A, Brooks LD*, et al.* An integrated map of genetic variation from 1,092 human genomes. *Nature* 2012;491:56-65. doi:10.1038/nature11632

4 Karczewski KJ, Francioli LC, Tiao G*, et al.* The mutational constraint spectrum quantified from variation in 141,456 humans. *Nature* 2020;581:434-43. doi:10.1038/s41586-020-2308-7

5 Yeo G and Burge CB. Maximum entropy modeling of short sequence motifs with applications to RNA splicing signals. *J Comput Biol* 2004;11:377-94. doi:10.1089/1066527041410418

6 Reese MG, Eeckman FH, Kulp D*, et al.* Improved splice site detection in Genie. *J Comput Biol* 1997;4:311-23. doi:10.1089/cmb.1997.4.311

7 Shapiro MB and Senapathy P. RNA splice junctions of different classes of eukaryotes: sequence statistics and functional implications in gene expression. *Nucleic Acids Res* 1987;15:7155-74.

8 Li H and Durbin R. Fast and accurate short read alignment with Burrows-Wheeler transform. *Bioinformatics* 2009;25:1754-60. doi:10.1093/bioinformatics/btp324

9 Plagnol V, Curtis J, Epstein M*, et al.* A robust model for read count data in exome sequencing experiments and implications for copy number variant calling. *Bioinformatics* 2012;28:2747-54. doi:10.1093/bioinformatics/bts526

10 Turnbull C, Scott RH, Thomas E*, et al.* The 100 000 Genomes Project: bringing whole genome sequencing to the NHS. *Bmj* 2018;361:k1687. doi:10.1136/bmj.k1687

11 Conrad DF, Pinto D, Redon R*, et al.* Origins and functional impact of copy number variation in the human genome. *Nature* 2010;464:704-12. doi:10.1038/nature08516

12 Ye K, Guo L, Yang X*, et al.* Split-Read Indel and Structural Variant Calling Using PINDEL. *Methods Mol Biol* 2018;1833:95-105. doi:10.1007/978-1-4939-8666-8_7

13 El-Gazzar A, Mayr JA, Voraberger B*, et al.* A novel cryptic splice site mutation in COL1A2 as a cause of osteogenesis imperfecta. *Bone Rep* 2021;15:101110. doi:10.1016/j.bonr.2021.101110

14 Rentzsch P, Schubach M, Shendure J*, et al.* CADD-Splice-improving genome-wide variant effect prediction using deep learning-derived splice scores. *Genome Med* 2021;13:31. doi:10.1186/s13073-021-00835-9

15 Ioannidis NM, Rothstein JH, Pejaver V*, et al.* REVEL: An Ensemble Method for Predicting the Pathogenicity of Rare Missense Variants. *Am J Hum Genet* 2016;99:877-85. doi:10.1016/j.ajhg.2016.08.016

16 Adzhubei IA, Schmidt S, Peshkin L*, et al.* A method and server for predicting damaging missense mutations. *Nat Methods* 2010;7:248-9. doi:10.1038/nmeth0410-248

17 Ng PC and Henikoff S. SIFT: Predicting amino acid changes that affect protein function. *Nucleic Acids Res* 2003;31:3812-4.

18 Melé M, Ferreira PG, Reverter F*, et al.* Human genomics. The human transcriptome across tissues and individuals. *Science* 2015;348:660-5. doi:10.1126/science.aaa0355
